# A riot of rhythms: neuronal and glial circadian oscillators in the mediobasal hypothalamus

**DOI:** 10.1186/1756-6606-2-28

**Published:** 2009-08-27

**Authors:** Clare Guilding, Alun TL Hughes, Timothy M Brown, Sara Namvar, Hugh D Piggins

**Affiliations:** 1 Faculty of Life Sciences, University of Manchester, Manchester, UK

## Abstract

**Background:**

In mammals, the synchronized activity of cell autonomous clocks in the suprachiasmatic nuclei (SCN) enables this structure to function as the master circadian clock, coordinating daily rhythms in physiology and behavior. However, the dominance of this clock has been challenged by the observations that metabolic duress can over-ride SCN controlled rhythms, and that clock genes are expressed in many brain areas, including those implicated in the regulation of appetite and feeding. The recent development of mice in which clock gene/protein activity is reported by bioluminescent constructs (luciferase or luc) now enables us to track molecular oscillations in numerous tissues *ex vivo*. Consequently we determined both clock activities and responsiveness to metabolic perturbations of cells and tissues within the mediobasal hypothalamus (MBH), a site pivotal for optimal internal homeostatic regulation.

**Results:**

Here we demonstrate endogenous circadian rhythms of PER2::LUC expression in discrete subdivisions of the arcuate (Arc) and dorsomedial nuclei (DMH). Rhythms resolved to single cells did not maintain long-term synchrony with one-another, leading to a damping of oscillations at both cell and tissue levels. Complementary electrophysiology recordings revealed rhythms in neuronal activity in the Arc and DMH. Further, PER2::LUC rhythms were detected in the ependymal layer of the third ventricle and in the median eminence/pars tuberalis (ME/PT). A high-fat diet had no effect on the molecular oscillations in the MBH, whereas food deprivation resulted in an altered phase in the ME/PT.

**Conclusion:**

Our results provide the first single cell resolution of endogenous circadian rhythms in clock gene expression in any intact tissue outside the SCN, reveal the cellular basis for tissue level damping in extra-SCN oscillators and demonstrate that an oscillator in the ME/PT is responsive to changes in metabolism.

## Background

The coordinated daily regulation of cycles in rest and activity, food intake and metabolism are crucial for the optimal health of an individual [1-4]. In mammals this daily, or circadian, timekeeping is customarily attributed to the intrinsic activities of autonomous cellular clocks within the suprachiasmatic nuclei (SCN) of the hypothalamus [5,6]. Individual SCN cells sustain robust and synchronized endogenous rhythms in clock gene expression [7]. These enable the SCN to maintain a coherent rhythmic tissue output which underlies its basis as the master clock controlling daily rhythms in physiology and behavior [8-11]. Many peripheral cells and tissues also rhythmically express clock genes, the phases of which are coordinated throughout the organism by the SCN, which is itself entrained by environmental timing cues [12-14].

Unlike peripheral circadian pacemakers, there is only limited evidence of overt self-sustained circadian oscillations in the central nervous system, outside of the SCN [15-18]. Monitoring of reporter constructs such as luciferase (luc) driven by clock genes or their protein products has enabled the investigation of endogenous extra-SCN oscillators. Structures such as the arcuate nuclei of the hypothalamus (Arc), olfactory bulb and hippocampus show intrinsic tissue level circadian rhythms in *per1::luc*, and the olfactory bulb shows rhythms in neuronal firing *in vitro*, which likely contribute to daily alterations in the functioning of these tissues [15,19]. However, compared to the SCN, these rhythms damp rapidly and single cell resolution in intact tissues has yet to be achieved.

Given the emerging link between circadian regulation and metabolic function in health and disease [2,3,20-26], we sought to determine the presence and organization of endogenous neural oscillators in the mouse mediobasal hypothalamus (MBH), an area critically important in governing internal metabolic homeostasis [27,28]. The MBH encompasses the Arc, dorsomedial hypothalamus (DMH), ventromedial hypothalamus (VMH), median eminence (ME) and pars tuberalis (PT). All of these tissues have been implicated in biological timekeeping [29-32] and intense interest surrounds the DMH as a possible component of the SCN-independent food entrainable oscillator [33-36]. However, very little is known about the capability of cells in these regions to sustain circadian oscillations.

Using a highly sensitive microscopy system to visualize PER2::LUC expression and longitudinal monitoring of neural activity, we track endogenous circadian rhythms in single cells and tissues in multiple regions of the MBH. Collectively, our results provide the first description of the tissue organization of extra-SCN neural oscillators, indicate that these can be differentially reset by metabolic cues and highlight key mechanistic differences in cellular organization between the master SCN clock and downstream oscillators.

## Methods

### Animals and Feeding Paradigms

Adult male *mPer2Luc *knockin mice (PER2::LUC; [37]) from the University of Manchester breeding colony were group-housed under a 12 h light:12 h dark (LD) cycle with *ad libitum *access to standard lab chow (5%/20%/75% calories from fat, protein and carbohydrate respectively; Beekay Rat and Mouse Diet No.1, B&K Universal, Hull, UK) and water for at least two weeks prior to experimentation. Temperature was maintained at ~18°C and humidity at ~40%. All procedures were carried out in accordance with the UK Animals (Scientific Procedures) Act 1986.

Standard lab chow *ad libitum*-fed mice used for initial bioluminescence and electrophysiology studies were culled directly from group housing. For food deprivation (FD) studies, group-housed mice were starved for ~14 h; food was removed 15 min before lights off (ZT11.75) and mice were culled at ZT1.5-ZT2 the following morning. Time-matched, similarly group-housed, *ad libitum*-fed mice were used as controls.

For high-fat feeding studies (HFF), mice were singly housed and maintained on a diet providing 45% of calories from fat (35% from carbohydrate and 20% from protein; DIO series, D12451, Research Diets Inc., New Jersey, USA) for at least 11 wks. Age-matched, singly-housed mice fed an *ad libitum *standard diet were used as controls for the HFF part of the study.

### Culture Preparation

Mice were culled by cervical dislocation following halothane anesthesia (Concord Pharmaceuticals, Essex UK), at a range of times during the LD cycle, with the aid of night vision goggles during dark periods to prevent exposure of animals to light. Following removal, brains were cooled and moistened with ice cold Hank's Balanced Salt Solution (HBSS; Sigma, Poole, UK) supplemented with 0.035% sodium bicarbonate (Sigma), 0.01 M HEPES (Sigma) and 1 mg/ml penicillin-streptomycin (Gibco Invitrogen Ltd, Paisley, UK). 300 μm thick coronal brain slices were cut using a vibroslicer (Camden Instruments, Leicester, UK) and transferred to sterile tissue culture dishes (Corning Inc., New York, USA) in cold HBSS. Using a dissecting microscope and mouse brain atlas (Paxinos and Franklin, 2001), brain regions were identified and excised with a pair of scalpels to leave either intact bilateral MBH explants, containing DMH, VMH, Arc and ME/PT, or microdissected individual explants of either bilateral DMH, Arc complex (Arc/ME/PT) or SCN. In preliminary experiments, 300 μm thick bilateral MBH sections were taken from between -1.40 mm to -2.20 mm bregma [38]. Maximal PER2::LUC bioluminescence was observed in slices between -1.80 mm and -2.10 mm bregma, so all subsequent MBH explants were taken from this level. Excised tissue was cultured on interface-style Millicell culture inserts (PICMORG50, Millipore (U.K.) Ltd., Watford, UK) in glass coverslip-based culture dishes (Fluorodish, World Precision Instruments Ltd., Stevenage, UK) for bioluminescence imaging (see below) or standard 35 mm plastic-based cultures dishes (Corning, UK) for PMT recording. 1.2 ml (WPI dishes) or 1 ml (Corning dishes) of sterile culture medium (DMEM; Dulbecco's Modified Eagle's Medium (D-2902, Sigma) supplemented with 3.5 g/L D-glucose (Sigma); 0.035% sodium bicarbonate (Sigma); 10 mM HEPES buffer (Sigma); 1 mg/ml penicillin-streptomycin (Gibco); B27 (Invitrogen) or 5% fetal bovine serum (FBS; Gibco) and 0.1 mM luciferin (Promega, Southampton, UK) in autoclaved Milli-Q water) were used per culture. No differences were observed between cultures maintained in serum-containing medium (FBS) and serum-free (B27) medium (see additional file 1: Fig. S1). Dishes were sealed with a glass coverslip using autoclaved high-vacuum grease (Dow Corning Ltd., Coventry, UK) and transferred directly to the bioluminescence imaging systems or PMT incubators for bioluminescence recording.

### Bioluminescence Imaging

Initial imaging of bioluminescence from standard lab chow-fed mice was performed using a self-contained Olympus Luminoview LV200 luminescence microscopy system (Olympus, Japan) fitted with a cooled Hamamatsu ImageEM C9100-13 EM-CCD camera and 20 × 0.4 NA Plan Apo objective (Olympus). For FD studies, bioluminescence images were acquired using either the above system or an Andor Ikon-M 934 CCD camera on the LV200 platform.

Images were acquired consecutively for 2–14 days at 37°C in darkness. Gain and exposure times were kept constant for MBH cultures within the initial imaging part of the study (LV200 with Hamamatsu camera) to allow direct comparison of the levels of bioluminescence between brain regions in this area.

Images from the acquisition software were transferred to ImageJ (version 1.37a, NIH, USA) and combined into a series of 30 min or 1 h average projections. A region of interest tool was used to delineate discrete nuclei (DMH, VMH, Arc, ME/PT and ependymal cells of the 3rd ventricle) or single cells within these areas and assess relative bioluminescence over time.

### PMT Bioluminescence

Total bioluminescence was recorded for up to 14 days from individual brain slice cultures with photomultiplier tube assemblies (H8259/R7518P, Hamamatsu, Welwyn Garden City, UK) housed in a light tight incubator (Galaxy R+, RS Biotech, Irvine, Scotland) maintained at 37°C. Photon counts were integrated for 59 s every 1 min. Bioluminescence data were detrended by subtracting a 24 h running average from the raw data and smoothed with a 3 h running average.

### Tetrodotoxin, Forskolin and Potassium Chloride Treatment

To assess the contribution of sodium channel dependant action potentials to the generation and maintenance of bioluminescence rhythms in the MBH, explants were maintained with a voltage-gated sodium channel blocker, tetrodotoxin (TTX; 0.5 μM, Sigma), in the culture medium. Tissue viability following damping of bioluminescence rhythms (both in the presence and absence of TTX) was assessed by treatment of cultures with either the adenylate cyclase activator forskolin (10 μM, Sigma), or the depolarizing stimulus potassium chloride (KCl; 10 μM, Sigma) 5–8 days following culture. Treatments were performed as a complete medium change to fresh forskolin or KCl-containing culture medium, otherwise identical to initial DMEM based culture medium with or without TTX as appropriate.

### Slice Preparation for Extracellular Recording

Slices were prepared during the lights-on phase and maintained using methods similar to those described earlier [39]. Mice were culled by cervical dislocation and decapitation, the brain was removed and placed in 4°C artificial cerebrospinal fluid (aCSF: pH 7.4) of composition: NaCl 124 mM, KCl 2.2 mM, KH_2_PO_4 _1.2 mM, CaCl_2 _2.5 mM, MgSO_4 _1.0 mM, NaHCO_3 _25.5 mM, D-glucose 10 mM, ascorbic acid 1.14 mM. Coronal brain sections (350 μm thick), from the same rostral-caudal coordinates as slices used for bioluminescence imaging, were cut using a vibroslicer (Campden Instruments), transferred to the recording chamber and equilibrated for ~1 h before the start of electrophysiological experiments. Throughout the dissection procedure aCSF was bubbled with 95% O_2_/5% CO_2_.

### Extracellular Recordings

Slices were maintained at 34 ± 1°C in an submerged recording chamber (PDMI-2; Harvard apparatus, Eddenbridge, UK), continuously perfused with oxygenated aCSF at ~1.5 ml/min. Slices were transilluminated and visualized under a dissecting microscope, and micromanipulators were used to precisely guide electrode tips onto the ArcD, DMHc or VMH (see additional file 1: Fig. S2). Extracellular multiunit activity (MUA) was recorded from these regions for at least 48 h, using aCSF-filled suction electrodes constructed as previously described [40]. In some experiments MUA was recorded from two brain regions simultaneously using two separate electrodes (see additional file 1: Fig. S2). Multiunit signals were differentially amplified (× 20,000) and bandpass filtered (300–3000 Hz) via a Neurolog system (Digitimer, Welwyn Garden City, UK), digitized (25,000 Hz) using a micro 1401 mkII interface (Cambridge Electronic Design (CED), Cambridge, UK) and recorded on a PC running Spike2 version 6 software (CED).

Using Spike2 software, single unit activity was discriminated offline from these MUA recordings as previously described [39,40]. Briefly, single units were discriminated on the basis of waveform shape, principal components-based clustering, and the presence of a clear refractory period in an interspike interval histogram. Using these criteria we were able to successfully isolate up to four single units from each recording.

### Data Analysis

Molecular and electrophysiological rhythms were analyzed using curve fitting software (Clockwise, developed in house by Dr T. Brown) as previously described.) [41]. Initially, data were normalized so that they spanned a range of values between 100 and -100. The normalized data was then fitted with the equation Y = Asin(B(x+C)) using the Newton-Raphson iterative method, where A equaled the amplitude of the rhythm, B equaled the period in radians/h and C determined the phase. Initial values of A, B and C were estimated from the best fitting curve of a series of > 3000 standard curves with periodicities between 3–34 h and a range of different amplitudes and phasing. Significant rhythmic variation in the data was assessed by repeating the curve fitting procedure 1000 times using the same dataset, but with the order of observations randomized with respect to time.

#### Bioluminescence

Total bioluminescence (PMT) and processed bioluminescence image data from delineated discrete nuclei and single cells were assessed with Clockwise to determine the significance of circadian variation in PER2::LUC expression. Cultures were prepared at a range of times to assess whether the phase of rhythms was related to time of cull. Rayleigh vector plots indicated that the phase of peak PER2::LUC expression in the ArcD, ArcL and DMH (n = 12) were significantly correlated with time of cull (p < 0.00001; see additional file 1: Fig. S3). Consequently all graphed MBH data were aligned with 0 on the x axis as cull time, and the initial phase of rhythms calculated using the peak of the circadian oscillation in PER2::LUC expression during the interval between 24 and 48 h in culture. Period (peak-peak and trough-trough averaged), phase and amplitude (peak-trough 24–48 h after culture) were assessed manually by two experienced, independent researchers. Rate of damping, calculated as the number of cycles observed before bioluminescence levels reached the previously determined level of dark noise (± 10%), was similarly assessed. Where cultures showed obvious damping of the bioluminescence rhythm but had not fully damped by the end of data acquisition, the projected rate of damping was calculated. Period and phase measurements were subsequently confirmed with Clockwise and in all cases were found to be in close agreement with manually assessed data. Imaged MBH cultures were further assessed to determine the percentage of discriminated single cells expressing a significant circadian rhythm as determined by Clockwise. Paired and unpaired t-tests (Microsoft Excel) and one way ANOVA with Tukey *post hoc *or single degree of freedom *a priori *tests (SYSTAT version 10; SPSS, Chicago, IL; p < 0.05 required for significance) were used as appropriate to determine statistical significance. Cellular synchrony was assessed and visualized using Rayleigh analysis, raster plots (see additional file 1: Fig. S4) and cross correlations (El Temps; Dr. A. Díez-Noguera, Barcelona, Spain; and software designed in house by Dr. T. Brown).

## Results

Nuclei of the neuroendocrine system within the MBH control a wide array of physiological and behavioral functions, many of which display precise temporal cycles, often with a circadian or diurnal basis. To examine the endogenous circadian rhythmicity of multiple hypothalamic nuclei and their intra-tissue organization, we assessed clock gene expression in acute slice cultures of MBH and SCN prepared from adult PER2::LUC mice and neuronal activity *in vitro *using longitudinal electrophysiological recordings.

### The MBH expresses circadian rhythms in PER2::LUC bioluminescence and neural activity

**Figure 1:**
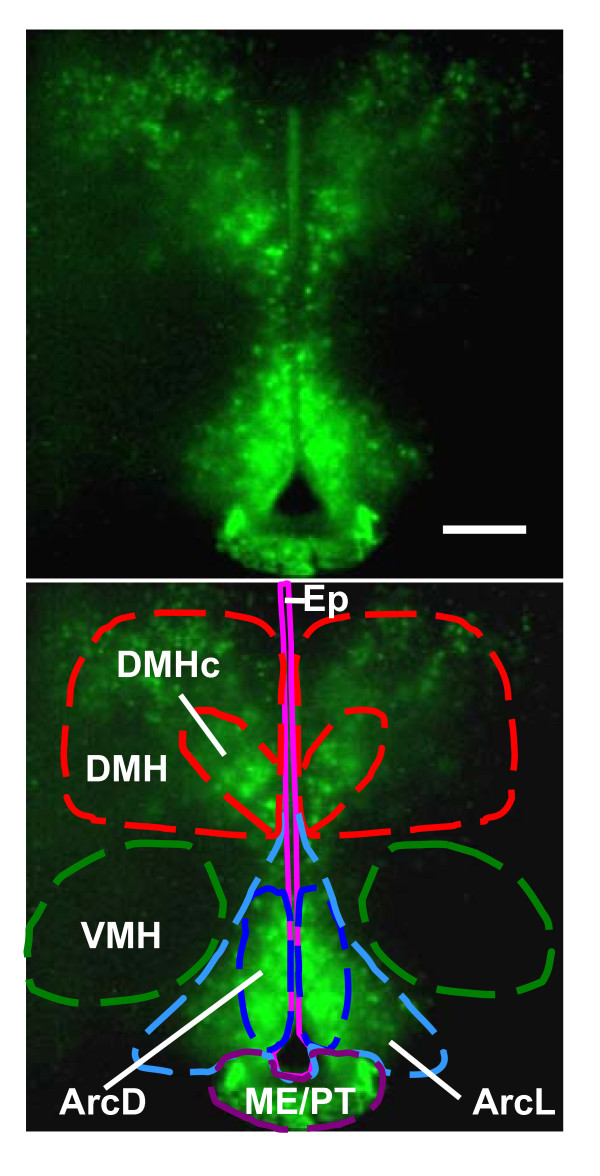
**PER2::LUC expression in the mediobasal hypothalamus**. EM-CCD image illustrating PER2::LUC bioluminescence in the dorsomedial hypothalamus (DMH), particularly in the pars compacta region (DMHc), in the lateral and dorsal arcuate (ArcL and ArcD), the median eminence/pars tuberalis (ME/PT) and the ependymal cell layer of the 3rd ventricle (Ep), but not in the ventromedial hypothalamus (VMH). Calibration bar 250 μM. Lower panel delineates nuclei of interest.

**Figure 2:**
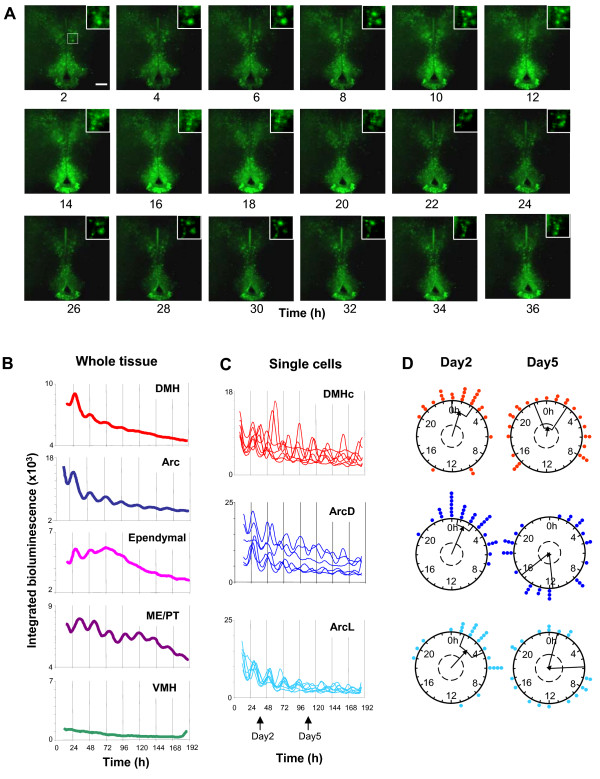
**Circadian rhythms of PER2::LUC expression in the MBH**. (**A**) EM-CCD images from an MBH slice (representative of 12 independent experiments) showing one and a half circadian cycles of PER2::LUC bioluminescence expression. Single cells can be discriminated in the DMH (inset) and Arc. Calibration bar 250 μm. (**B**) Plots of relative PER2::LUC expression integrated across delineated DMH, Arc, ependymal cell layer, ME/PT and VMH. Expression is circadian in all regions except the VMH. Note how tissue wide circadian expression damps at different rates in different tissues. (**C**) Plots of integrated bioluminescence for representative individual cells in the DMHc, ArcD and ArcL from the same slice. Individual cells are still rhythmic after 8 days in culture, yet the amplitude and synchrony of rhythms decreases over time. (**D**) Rayleigh vector plots showing phase clustering of cells in the DMHc, ArcD and ArcL at 2 days and 5 days following culture (days indicated in panel C). Circadian rhythms are initially synchronized in all areas (day 2: DMHc, p < 0.005; ArcD, p < 0.005; ArcL p < 0.05) but become desynchronized over the course of the experiment (day 5: all p > 0.05). Filled circles indicate the phase of individual cells. Direction of arrow indicates the mean phase vector, its length indicates the significance of phase clustering, with the surrounding box indicating the variance of phase. The inner broken line indicates the significance threshold of p = 0.05.

Whole MBH slice cultures were imaged in real time with an EM-CCD camera-equipped microscopy system. The coronal slice culture employed here provides a unique and novel opportunity to visualize a number of different oscillators in the same section. Within each slice, the DMH, VMH, Arc and ME/PT were visualized (Fig. 1). Continuous recordings of PER2::LUC activity were made from 12 slices for up to 14 days *in vitro*. All recordings revealed clear PER2::LUC expression in the DMH, primarily in the *pars compacta *region (DMHc), in the Arc, ME/PT and in the ependymal cell layer of the third ventricle, but surprisingly, not in the VMH. PER2::LUC expression showed significant circadian rhythmicity in all regions in which it was visualized (Fig. 2; also see additional files 2 and 3: Movies 1 and 2; significance determined by Clockwise rhythm analysis software; p < 0.05 required for significance). Further, single cells were readily discriminated in both the DMH and Arc (Fig. 2A and 2C). Cultures were prepared at a range of times across the LD cycle to assess whether the phase of rhythms was related to time of cull. Rayleigh vector plots indicated that, unlike in the SCN, the phase of peak PER2::LUC expression in the Arc and DMH was significantly correlated with time of cull (p < 0.00001; see additional file 1: Fig. S3 and Methods section).

As an additional measure of intrinsic timekeeping capabilities we performed long-term electrophysiological recordings from the DMHc, the dorsal Arc (ArcD) and the VMH. Circadian rhythms in population and single cell firing were observed in the ArcD. Weak population rhythms were found in the DMHc, and only rarely in the VMH. Detailed results from each region of the MBH are reported below.

### Arcuate

#### Circadian rhythms in PER2::LUC activity

The Arc exhibited circadian cycles of PER2::LUC bioluminescence in all MBH slices (n = 12; Figs. 1 and 2; also see additional files 2 and 3: Movies 1 and 2), with rhythmicity sustained for up to 8 days (mean ± SEM: 5.2 ± 0.6 days; Fig. 2B, Table 1). The average period of oscillations in the Arc was 23.1 ± 0.3 h (mean ± SEM; Table 1). In total, 226 single cells were visualized in the Arc. We segregated the Arc into its dorsal (ArcD) and lateral (ArcL) regions for single cell analysis (Fig. 1). Within the ArcD 129 cells were discriminated, 89.1% of which were rhythmic, while of 97 cells identified in the ArcL, 67.0% displayed rhythmicity; a significantly smaller percentage than in the ArcD (p < 0.05, paired t-test; Table 1). In addition to the increased proportion of rhythmic cells in the ArcD versus ArcL, the amplitude of ArcD rhythms was also significantly higher p < 0.05, paired t-test; Fig. 2C, Table 1). The period of oscillations did not differ between cells in different subdivisions of the Arc (p > 0.05, paired t-test; Table 1; also see additional file 1: Fig. S5). Peak PER2::LUC expression in the whole Arc was observed 28.6 ± 0.3 h after cull. Oscillations in single cells in the ArcD peaked at 28.6 ± 0.6 h after cull, and in the ArcL peaked at 28.5 ± 0.6 h after cull (Table 1). Rayleigh tests were used to investigate cellular synchrony two days after cull in the Arc. In all twelve slices in which rhythmic cells were found, significant phase clustering was observed in the ArcD and ArcL (Fig 2D; p < 0.005 and p < 0.05, respectively).

**Table 1:** Bioluminescence data from control animals

Tissue	n	% Rhythmic	Period (hours)	Phase (hours after cull)	Amplitude (arbitrary units)	Rate of Damping (days)
**Camera**						
DMH (whole)	12	100	21.9 ± 0.8	28.6 ± 0.6	2247 ± 775	3.0 ± 0.4
DMH (single cells)	131	81.7	22.4 ± 0.3	28.4 ± 1.3	2483 ± 400	†
Arc (whole)	12	100	23.1 ± 0.3	28.6 ± 0.3	3658 ± 1128	5.2 ± 0.6
Dorsal Arc (single cells)	129	89.1	22.6 ± 0.6	28.6 ± 0.6	5805 ± 2221	†
Lateral Arc (single cells)	97	67.0	22.6 ± 0.4	28.5 ± 0.6	2854 ± 812	†
Ependymal	12	92	22.3 ± 0.5	30.4 ± 1.3	1168 ± 234	3.6 ± 0.5
ME/PT	12	100	23.3 ± 0.4	31.3 ± 0.9	1901 ± 740	6.9 ± 1.0
SCN (whole)	4	100	24.1 ± 0.3	9.4 ± 0.8^	14622 ± 5835^#^	∞
SCN (single cells)	40	97.5	23.6 ± 0.1	8.0 ± 0.2^	21444 ± 1297^#^	∞

**PMT**						
Whole MBH	11	100	22.8 ± 0.6	29.4 ± 0.7	325 ± 61	3.3 ± 0.2
DMH	13	100	24.2 ± 0.5	30.6 ± 0.7	170 ± 21	2.6 ± 0.2
Arc/ME/PT	10	100	23.5 ± 0.6	30.5 ± 0.7	169 ± 31	3.1 ± 0.3
SCN	6	100	23.9 ± 0.2	9.8 ± 0.2^	5426 ± 1215	∞

^ phase for SCN cultures is in Zeitgeber time, not time from cull, as SCN rhythms are not reset by cull/culture procedure.

† not all single cells had damped completely by the end of imaging; ~80% of cells were still clearly oscillating after one week.

^# ^values not directly comparable to data for MBH regions as different detection settings were used to image SCN cultures due to their much higher levels of bioluminescence (see experimental procedures/results).

#### The ArcD expresses circadian rhythms in neural activity

**Figure 3:**
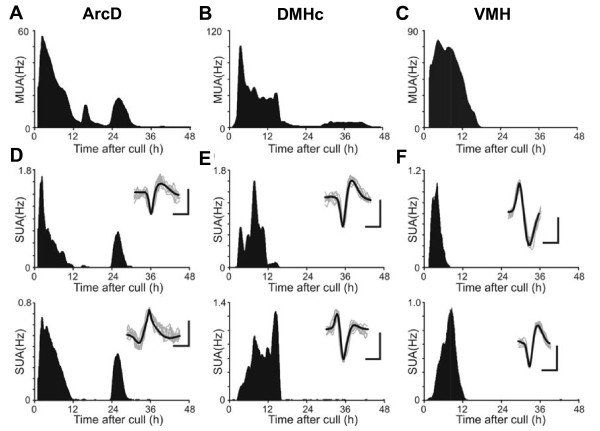
**Temporal patterns of electrical activity in mediobasal hypothalamic nuclei**. Mediobasal hypothalamic nuclei exhibited a continuum of temporal profiles in electrical activity. Recordings of ArcD exhibited robust multiunit (MUA; **A**) and single unit activity (SUA; **D**) rhythms. MUA rhythms recorded from the DMHc (**B**) damped more rapidly than in the ArcD, while SUA profiles typically exhibited a single peak (**E**). In contrast, the ventromedial hypothalamus rarely sustained detectable MUA (**C**) or SUA (**F**) for longer than 18 h. Inset traces in **D-F **indicate the average spike waveforms for each cell (thick black line) and 8 consecutive matching spikes (thin grey lines); scale bars represent 15 μV (vertical) and 1 ms (horizontal). All figures are representative examples from single slices of MBH. Abbreviations as in text.

Population and single cell electrophysiological activity were monitored in the ArcD for at least 48 hours *in vitro*. Clear circadian oscillations were detected in population discharge in 5 of 6 recordings (Fig. 3A; mean period 24.0 ± 0.7 h) with peak discharge occurring 19.0 ± 2.7 h after cull. From these five rhythmic recordings we discriminated the firing profiles of 12 individual neurons, of which 10 (83%) exhibited overt circadian rhythms (Fig. 3D; mean period: 23.9 ± 0.3 h), with peak firing occurring 18.8 ± 1.8 h after cull. In the remaining ArcD slice, multiunit firing displayed one clear peak then damped out. In this slice we were able to discriminate 4 distinct single units of which 3 were rhythmic, but with differing periods (16.0–22.3 h), explaining the lack of rhythmicity in the multiunit profile. Rayleigh analysis of the timing of multi and single unit peak firing showed that there was no significant clustering of peak cellular electrical discharge in relation to either time of cull or lighting schedule under which the animal was housed (p > 0.05, data not shown).

### Dorsomedial hypothalamus

#### Circadian rhythms in PER2::LUC activity

PER2::LUC bioluminescence was detected in the DMH, localized primarily in the DMHc region (Figs. 1 and 2; also see additional files 2 and 3: Movies 1 and 2). In all imaged MBH slices, DMH PER2::LUC expression displayed significant circadian rhythmicity, sustained on average for 3.0 ± 0.4 days (Fig. 2A and 2B, Table 1). The average period of oscillations in the DMH was 21.9 ± 0.8 h (Table 1, Fig. 2). In total, 131 single cells were detected in the DMH; these were mainly located in the ventromedial area of the DMHc (Figs. 1 and 2). Of the cells discriminated, 81.7% were rhythmic with an average period of 22.4 ± 0.3 h (Table 1; also see additional file 1: Fig. S5). On average, oscillations in the whole DMH peaked at 28.6 ± 0.6 h after cull and oscillations in single cells peaked 28.4 ± 1.3 h after cull (Table 1). Rayleigh tests for cell synchrony two days after cull revealed significant phase clustering in all eight slices in which single cells could be discriminated (p < 0.005; Fig. 2D), indicating a tight synchrony of molecular timekeepers.

#### The DMHc expresses weak circadian rhythms in neural activity

Circadian rhythms in multiunit discharge in the DMHc damped more rapidly than those detected in the ArcD. In total, 3/6 slices showed 2 distinct peaks in multiunit activity (Fig. 3B; mean period 23.2 ± 1.7 h), although the peaks on day 2 were consistently of lower amplitude than observed in the ArcD. The remainder exhibited single peaks. Peak discharge occurred 14.0 ± 1.6 h after cull. We discriminated 14 individual neurons from the DMH multiunit recordings (Fig. 3E). All but one of these cells exhibited a single peak in activity (14.2 ± 1.6 h after cull), with the remaining cell exhibiting a second peak defining a weak ~24 h firing rate rhythm. As with our ArcD recordings, timing of firing rate peaks appeared random with respect to cull time or lighting schedule (data not shown).

### Ventromedial hypothalamus

No overt PER2::LUC expression was seen in the VMH in any MBH slice (Figs. 1 and 2), and measures of background bioluminescence revealed no rhythm of PER2::LUC bioluminescence in this region (Fig. 2A and 2B). Multiunit recordings of spontaneous discharge in the VMH (Fig. 3C) consistently reached peak values within the first 12 h of the recording session (7.9 ± 1.4 h after cull, n = 6), and in 5/6 slices exhibited a single peak in multiunit activity, with firing decaying to very low levels for the remainder of the recording session. One slice showed a low amplitude firing rhythm with a periodicity close to 24 h. We discriminated 17 single units from these VMH multiunit recordings (Fig. 3F). Only one of these single units exhibited detectable circadian rhythmicity, while the remaining cells displayed only a single peak in activity that occurred near the beginning of the recording session (7.4 ± 1.1 h after cull). These results indicate that rhythms in clock gene expression and neuronal activity previously recorded in the VMH *in vivo *[42,43] are most likely driven by pacemakers external to the VMH. Indeed, lesions of the SCN abolish the multiunit cell-firing activity recorded in the rat VMH *in vivo *[44,45]. This shows that the VMH is a slave oscillator, capable of displaying circadian rhythmicity *in vivo*, but only under the influence of a master oscillator.

### Median eminence/pars tuberalis

PER2::LUC expression was observed in the ME/PT in all MBH slices and was often clearly delineated in the PT region. Circadian rhythmicity in PER2::LUC expression was observed in all slices and was sustained for ~1 week (mean ± SEM: 6.9 ± 1.0; Fig. 2B, Table 1). No single cells could be discriminated in this region. The average period of the oscillations from all slices was 23.3 ± 0.4 h and peak PER2::LUC expression was observed 31.3 ± 0.9 h after cull (Table 1).

### Ependymal cell layer

Ependymal cells are epithelial glia that line the central cavities of the brain and are constituents of the walls of the ventricles. PER2::LUC expression was observed in the ependymal cell layer surrounding the third ventricle (Figs. 1 and 2). PER2::LUC bioluminescence in this region displayed circadian rhythmicity in 11/12 MBH slices (92%), typically sustained for ~3 days (mean ± SEM: 3.6 ± 0.5; Fig. 2B, Table 1). PER2::LUC expression generally appeared after the initial first peak of the Arc and DMH and appeared to travel bi-directionally: down from the top of the third ventricle, and up from the base of the ME in a wave like pattern (see additional files 2 and 3: Movies 1 and 2). No single cells could be discriminated in this layer. We assessed the rhythmicity of the dorsal region of the ependymal cell layer since it was difficult to clearly distinguish the ventral ependymal layer from the surrounding Arc. The average period of the oscillations from rhythmic slices was 22.3 ± 0.5 h and peak PER2::LUC expression was observed 30.4 ± 1.3 h after cull (Fig. 2, Table 1). Our data from this area provides the first evidence of endogenous circadian rhythms in hypothalamic glial cells and extends the knowledge of non-neuronal oscillators in the CNS [46].

### Circadian rhythms of PER2::LUC expression imaged in the SCN

**Figure 4:**
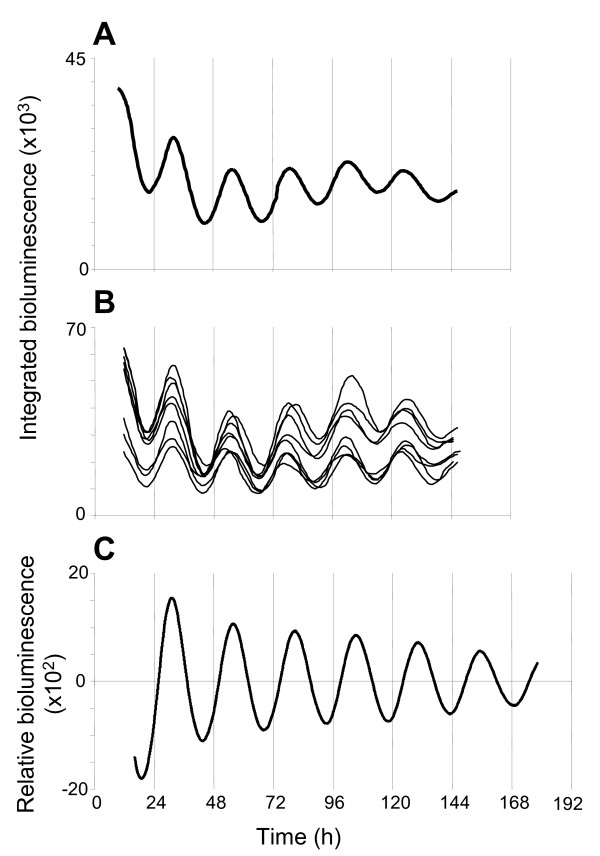
**Circadian rhythms of PER2::LUC expression in the SCN**. **(A) **Plot of relative PER2::LUC bioluminescence integrated across the whole SCN, imaged with an EM-CCD camera. **(B) **Plots showing circadian rhythms of PER2::LUC bioluminescence for representative individual cells from the slice in (A). **(C) **Detrended PMT recording of total PER2::LUC bioluminescence from an SCN slice.

For comparison with the above recordings of bioluminescence rhythms in MBH nuclei, we also assessed circadian variation of PER2::LUC expression in the SCN. Cultures cut from mid rostro-caudal levels of the SCN (n = 4) all expressed clear, high amplitude oscillations in PER2::LUC bioluminescence with an average period of 24.1 ± 0.3 h, and peak PER2::LUC expression was observed at ZT9.4 ± 0.8 (Fig. 4A; Table 1). Bioluminescence imaged from SCN cultures for up to 6 days showed no signs of significant damping over this timescale (Fig. 4A; Table 1). Individual PER2::LUC expressing cells were readily identifiable in the SCN (Fig. 4B); of 40 cells selected at random, 39 were rhythmic (97.5%), expressing a mean period of 23.6 ± 0.1 h (range: 22.7 h–25 h; see additional file 1: Fig. S5), with bioluminescence peaking at ZT8.0 ± 0.2. Cells within the SCN were significantly synchronized following two days in culture, and maintained this high degree of synchrony after five days in culture (Rayleigh plots, both p < 0.00001).

### Micro-dissected Arc complex and DMH maintain intrinsic rhythmicity

**Figure 5:**
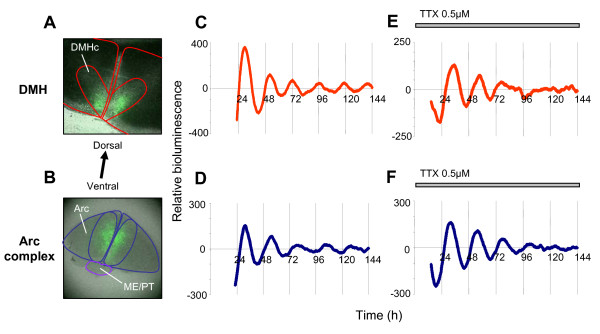
**Micro-dissected Arc complex and DMH maintain circadian rhythms in PER2::LUC expression in normal and TTX containing media**. EM-CCD images of micro-dissected Arc complex (Arc/ME/PT) (**A**) and DMH (**B**) captured before total bioluminescence was recorded in photomultiplier tubes (PMTs). (**C**; Arc complex) and (**D**; DMH) are detrended PMT recordings of relative PER2::LUC bioluminescence (counts per minute) emitted from the slices shown in (**A**) and (**B**). Inhibition of sodium channel dependent action potentials with TTX (0.5 μM) in micro-dissected Arc complex (**F**) and DMH (**E**) does not inhibit circadian rhythms in PER2::LUC expression.

To assess whether the rhythmicity in the Arc complex (Arc/ME/PT) and DMH were dependent on interconnections between these nuclei preserved in whole slice cultures, and to further determine the circadian characteristics of these tissues, we performed long term analysis of PER2::LUC expression recorded in photomultiplier tube assemblies (PMTs); firstly from slice cultures consisting of a coronal section of intact MBH, then from micro-dissected, independently cultured, Arc complex and DMH (Fig. 5A and 5B; Table 1).

Intact MBH slice cultures were initially imaged on the EM-CCD camera to confirm the location of PER2::LUC expression in the Arc, ME/PT and DMH, and then placed in PMTs. All intact MBH slices (n = 11) were rhythmic though rhythms had damped by 3.3 ± 0.2 days after culture (Table 1). All micro-dissected DMH (n = 13) and Arc complex (n = 10) cultures were rhythmic (Fig. 5C and 5D, Table 1). Circadian oscillations in both regions damped more rapidly than in the intact MBH, maintaining rhythms for 2.6 ± 0.2 days in the DMH and 3.1 ± 0.3 days in the Arc complex. The amplitudes of oscillations in both micro-dissected DMH cultures (170 ± 21, relative bioluminescence) and Arc complex (169 ± 31) were significantly lower than that of intact MBH cultures (325 ± 61; both p < 0.05; ANOVA with *a priori *single degree of freedom comparisons; Table 1), though did not differ to each other (p > 0.05).

PMT recordings of PER2::LUC bioluminescence expression in the SCN were assessed for comparison with the MBH. All cultures (n = 6) were rhythmic with a mean period of 23.9 ± 0.2 h. Peak bioluminescence activity was not correlated to time of cull, as in the MBH but was instead related to ZT, peaking at ZT9.8 ± 0.2 (Fig. 4C; Table 1). Oscillations of PER2::LUC bioluminescence in the SCN were of significantly higher amplitude than either micro-dissected DMH or Arc complex, or intact MBH cultures (SCN mean amplitude: 5426 ± 1215, relative bioluminescence; all p < 0.0001; ANOVA with *a priori *single degree of freedom comparisons; Table 1). PMT recordings of SCN cultures were maintained for up to 7 days at which time oscillations showed no significant signs of damping (Fig. 4C).

### PER2 rhythms in MBH nuclei are independent of TTX-sensitive sodium channel dependent action potentials

**Figure 6:**
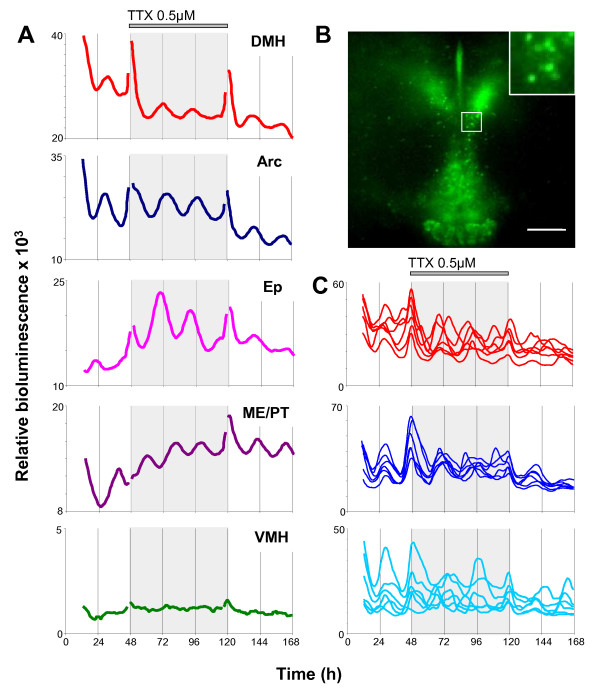
**Circadian rhythms of PER2::LUC expression continue in the presence of TTX**. **(A) **Representative example of total bioluminescence integrated across delineated DMH, Arc, ependymal cell layer (Ep), ME/PT and VMH in an intact MBH slice culture imaged on an EM-CCD camera. All areas except the VMH are initially rhythmic in control medium. After 2 days, the slice was treated with 0.5 μM TTX for 3 days before return to control medium, grey shading represents TTX in the culture medium. All previously rhythmic areas sustained rhythmicity in the presence of TTX. (**B**) EM-CCD image showing PER2::LUC bioluminescence expression in an MBH cultured with 0.5 μM TTX. Single cells can be discriminated in the DMH (inset) and Arc. Calibration bar 250 μm. (**C**) Plots showing integrated bioluminescence for representative individual cells in DMHc, ArcD (dark blue) and ArcL (light blue). Individual cellular rhythms continue in the presence of TTX.

To assess the autonomy of molecular rhythms in the MBH and its component nuclei, we impaired synaptic communication between cells by inhibition of sodium channel dependent action potentials with tetrodotoxin (TTX). 0.5 μM TTX, a dose we showed to completely inhibit neuronal firing in the MBH (see additional file 1: Fig. S6), does not inhibit PER2::LUC bioluminescence rhythms in the intact MBH (n = 6), DMH (n = 7) or Arc complex (n = 6) as assessed in PMTs. None of the parameters examined here (period, peak phase, amplitude or rate of damping), in the MBH or its constituent nuclei, were significantly affected by culture in TTX-containing medium (all p > 0.05; ANOVA with *a priori *single degree of freedom comparisons; Fig. 5E and 5F). When examined on the EM-CCD camera as part of intact MBH cultures, sustained circadian rhythmicity in the presence of TTX was observed in 6/6 Arc, 6/6 DMH, 5/6 ependymal cell layer and 6/6 ME/PT slice cultures. Single cells discriminated in the ArcD, ArcL and DMHc, maintained circadian rhythms in the presence of TTX (Fig. 6), indicating that individual cells have intrinsic pacemaking properties.

### Forskolin re-synchronizes individual cellular rhythms in DMH and Arc, reviving damped rhythms

The damping of circadian rhythms in the MBH and its component nuclei over time could be due to deteriorating tissue health, or result from the different dynamic properties of oscillators in this region. Forskolin, an activator of adenylate cyclase, was used to assess whether rhythms could be restarted in damped tissue. Forskolin treatment reinstated overt rhythms in all microdissected Arc (n = 10) and DMH (n = 10) cultures, as well as in intact MBH tissue (n = 10). In DMH cultures, rhythms recovered to at least the levels seen at the beginning of the recording and in the Arc complex, rhythms were revived with significantly higher amplitude than originally observed (p < 0.03, paired t-test). The effects of forskolin stimulation persisted when the culture medium also contained 0.5 μM TTX, indicating this action to be independent of sodium-dependent action potentials (see additional file 1: Fig. S7). These results demonstrate that damping of circadian rhythms was not due to deteriorating health of cultures, but to the damping of oscillations in individual cells and/or the desynchronization of independent oscillators in a cell population.

To investigate the cellular basis of damping rhythms we imaged MBH cultures on an EM-CCD camera and assessed the synchrony of peak phase and amplitude of oscillations for individual cellular rhythms at different times before and after treatment with forskolin (n = 3). The phase of peak PER2::LUC bioluminescence was synchronized 2 days after culture for cells in the ArcD, ArcL and DMHc (Rayleigh analysis, p < 0.05–p < 0.005; Fig. 2D; also see additional files 2 and 3: Movies 1 and 2). After 5 days in culture, cells in all regions had become desynchronized (Rayleigh analysis, p > 0.05 for all regions) and the amplitude of individual cellular rhythms in the ArcL and DMH had significantly reduced (p < 0.0001 and p < 0.005 respectively; Fig. 2C and 2D) while the amplitude of cellular rhythms in the ArcD had reduced, though not significantly (p > 0.05). This is reflected in the damped circadian bioluminescence emissions when integrated across the entire DMH or Arc (Fig. 2B). Thus, it appears that dampening tissue oscillations are due to a combination of progressive desynchronization of cellular rhythms and damping of individual cellular oscillators.

**Figure 7:**
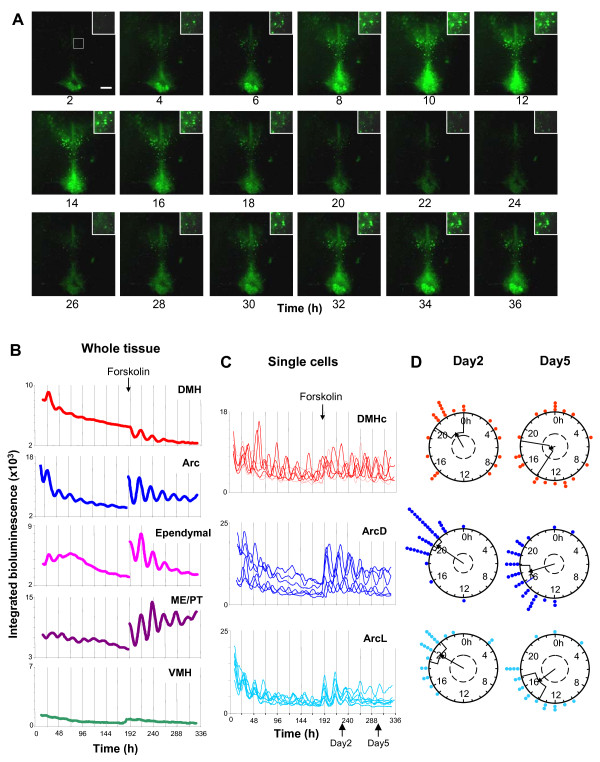
**Circadian rhythms of PER2::LUC expression are revived by forskolin treatment**. (**A**) EM-CCD images from the MBH slice depicted in Fig. 2 showing one and a half circadian cycles of PER2::LUC bioluminescence expression following addition of 10 μM forskolin to the culture medium. Single cells can be discriminated in the DMH (inset) and Arc. Calibration bar 250 μm. (**B**) Plots of relative PER2::LUC expression integrated across delineated DMH, Arc, ependymal cell layer, ME/PT and VMH. Circadian rhythms in all regions except the VMH are revived, and in the Arc, ME/PT and ependymal cell layer are potentiated with respect to initial amplitude. (**C**) Circadian rhythms in six representative individual cells in the DMHc, ArcD and ArcL are resynchronized. (**D**) Rayleigh vector plots showing phase clustering of cells in the DMHc, ArcD and ArcL at 2 days and 5 days following forskolin treatment (days indicated in panel **C**). Circadian rhythms are initially resynchronized in all areas (day 2: DMHc, p < 0.05; ArcD, p < 0.00001; ArcL p < 0.00001). Five days after forskolin treatment cells in the ArcD and ArcL are still synchronized (ArcD, p < 0.00001; ArcL p < 0.001). This continued synchrony is reflected in the sustained circadian rhythm in the Arc at day 5 after forskolin, when signal is integrated across the whole Arc (**B**). In contrast, individual cells in the DMHc become desynchronized by 5 days after forskolin (p > 0.05), which is reflected in the arrhythmicity in the whole DMH (**B**) at this time.

**Figure 8:**
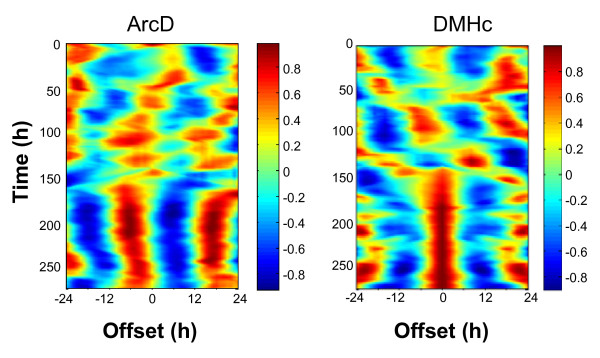
**Forskolin synchronizes cellular oscillators in the MBH**. Plots showing cross correlations between pairs of oscillating cells in the ArcD (left) and DMHc (right). Correlations were calculated from raw data using a moving window (duration 48 h) with the test cell shifted in time between -24 and +24 hours. The color scale indicates the strength of correlation at any given point with 1 (dark red) indicating perfect correlation and -1 (dark blue) perfect anti-correlation. Initially the cells shown were weakly synchronized (ArcD) or exhibited different circadian periods (DMHc). After addition of forskolin at time 150 h, the periods of cellular oscillators synchronized (indicated by stronger vertical banding) and adopted a stable phase relationship either in phase (DMHc) or with one cell phase leading the other by ~6 h (ArcD).

Forskolin (10 μM) treatment resynchronized individual cellular rhythms in all regions (Figs. 7 and 8; also see additional file 1: Fig. S4; p < 0.00001 for ArcD and ArcL, p < 0.05 for DMH: Rayleigh plots 2 days after treatment) and increased amplitude (p < 0.0001 for ArcD and ArcL, p < 0.05 for DMH; ANOVA with *a priori *single degree of freedom comparisons; Fig. 7). In contrast to the results in control culture medium, 5 days after forskolin treatment, cells in the ArcD and ArcL remained synchronized (p < 0.00001 and p < 0.001 respectively; Fig. 7C and 7D), however cells in the DMHc were once again desynchronized at this time (p > 0.05; Fig. 7C and 7D). These data correlate with the rate of damping seen at the whole tissue level, where the Arc maintained coherent rhythms for at least five days after forskolin treatment, while the DMH had become damped after three (Fig. 7B; Table 1). Despite this difference in the maintenance of cellular synchrony, 5 days following forskolin treatment the amplitude of individual cellular oscillations in all three regions had significantly damped once again (all p < 0.0001 vs. amplitude at day 2 following forskolin; all p > 0.05 vs. amplitude at day 5 in normal medium, before forskolin treatment; Fig. 7C).

### Food deprivation alters the phase of peak PER2::LUC in the ME/PT

**Figure 9:**
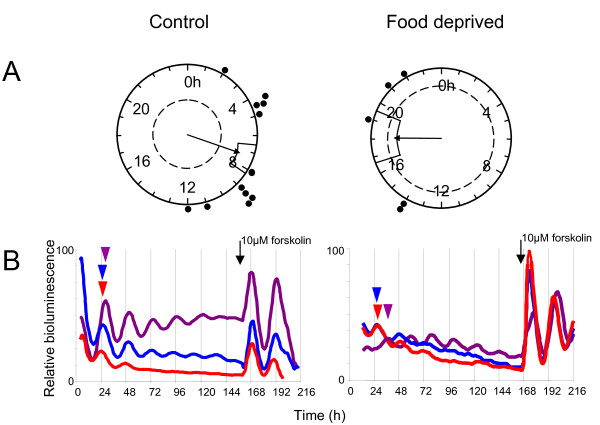
**The phase of peak PER2::LUC expression is altered in the ME/PT of food deprived mice**. (**A**) Rayleigh vector plots showing the phase of peak PER2::LUC expression *in vitro*, calculated as the time of peak bioluminescence after cull of animal, in the ME/PT of food deprived and control animals. In control animals the phase was significantly correlated with time of cull, peaking on average 31.3 ± 0.9 h after cull (n = 12) versus 42.4 ± 2.3 h in food deprived animals (n = 5, p < 0.001). (**B**) Lower panels highlight the differences in phase of PER2::LUC expression in neuronal tissue of the MBH in representative control and food deprived mice. Time of peak PER2::LUC is indicated by colored arrowheads; Arc (red), DMH (blue) and the ME/PT (purple). Note the altered peak phase of the ME/PT in the food deprived mouse.

Having demonstrated circadian oscillations in brain regions associated with feeding and metabolic behaviors we sought to investigate whether alterations in feeding regimes would affect rhythms in the MBH. A ~14 hour food deprivation paradigm resulted in an average body weight loss of 9.5% (25.71 g initial weight, vs. 23.27 after food deprivation, n = 13, p < 0.0001). Using PMT luminometry, a significant difference in the phase of peak PER2::LUC bioluminescence in the Arc complex was observed in food deprived versus time matched control animals (35. 8 ± 2.1 h vs. 31.3 ± 0.8 h respectively; p < 0.05, Table 2). No other circadian parameters were altered by overnight fast (Table 2). Detailed analysis of MBH slice cultures by photovideomicroscopy revealed this difference in phase to be specific to the ME/PT, which peaked in control animals at 31.3 ± 0.9 h after cull versus 42.4 ± 2.3 h in food deprived animals (p < 0.001, Fig. 9, Table 3). The Arc itself, along with the DMH and ependymal cell layer, maintained a normal phase with regards to control animals (Tables 1 and 3). Food deprivation did not induce rhythms in the VMH. Damped PER2::LUC expression in tissue explants from food deprived (and control mice) was consistently restarted by the addition of KCl, a potent depolarizing stimulus, to the culture medium (additional file 1, Fig. S8), similar to the response observed in the SCN pacemaker [8].

**Table 2:** PMT data from food deprived animals and age and time matched control

Animal/Tissue	n	% Rhythmic	Period (hours)	Phase (hours after cull)	Amplitude (arbitrary units)	Rate of Damping (days)
**Food deprived animals**						
Whole MBH	5	100	22.9 ± 0.1	34.2 ± 1.9	257 ± 73	6.5 ± 1.6
DMH	11	100	24.9 ± 0.6	30.7 ± 1.2	183 ± 38	2.4 ± 0.4
Arc/ME/PT	11	100	23.9 ± 0.6	35.8 ± 2.1*	181 ± 49	4.5 ± 1.2
SCN	9	100	24.6 ± 0.4	9.4 ± 0.8^	3701 ± 758	∞

**Controls**						
Whole MBH	5	100	23.7 ± 0.6	31.0 ± 1.6	239 ± 53	5.1 ± 0.6
DMH	8	100	23.8 ± 1.0	29.8 ± 0.4	157 ± 83	1.9 ± 0.4
Arc/ME/PT	8	100	23.4 ± 0.6	31.3 ± 0.8	191 ± 37	4.8 ± 0.5
SCN	5	100	24.3 ± 0.09	9.0 ± 0.5^	3882 ± 672	∞

^ phase for SCN cultures is in Zeitgeber time, not time from cull, as SCN rhythms are not reset by cull/culture procedure.

* p < 0.05 vs control animals

**Table 3:** Camera data from food deprived animals.

Tissue	n	% Rhythmic	Period (hours)	Phase (hours after cull)	Rate of Damping (days)
DMH (whole)	7	100	21.8 ± 0.4	30.2 ± 4.5	2.3 ± 0.5
DMH (single cells)	61	83.8	22.5 ± 0.6	34.6 ± 2.1	†
Arc (whole)	7	100	22.3 ± 0.4	30.7 ± 2.4	4.1 ± 0.4
Arc (single cells)	88	90.0	22.3 ± 0.2	33.6 ± 2.0	†
Ependymal	7	71.4	22.3 ± 0.5	30.4 ± 1.3	4.1 ± 1.4
ME/PT	6	85.7	21.8 ± 0.3	42.4 ± 2.3*	5.7 ± 1.0

Comparisons of amplitude between slices were not possible since more than one type of camera system was used.

* p < 0.001 vs control animals (cf Table 1)

† not all single cells had damped completely by the end of imaging.

### High-fat feeding does not alter PER2::LUC rhythms in the MBH or SCN

High-fat feeding (HFF) resulted in an average body weight gain of 72% (20.1 ± 0.6 g initial weight, to 34.5 ± 1.1 g after HFF, n = 6) over the course of the experiment, compared to a 17% increase in age matched control animals (21.1 ± 0.8 g to 24.8 ± 0.7 g; n = 4). There was, however, no overt difference in any circadian parameter in the SCN, Arc complex or DMH between tissue from HFF and control mice, as assessed by PER2::LUC luminometry (Table 4). These data indicate that changes in the hypothalamic expression of PER2 are not overtly involved in the etiology of circadian disruption associated with the ingestion of a diet rich in fat [21,47].

**Table 4:** PMT data from high-fat fed and controls

Animal/Tissue	n	% Rhythmic	Period (hours)	Phase (hours after cull)	Amplitude (arbitrary units)	Rate of Damping (days)
**HFF animals**						
DMH	5	100	24.9 ± 0.4	31.2 ± 0.9	125 ± 19	2.3 ± 0.2
ArcME/PT	5	80	24.3 ± 0.8	32.1 ± 0.6	255 ± 75	4.0 ± 0.5
SCN	5	100	24.9 ± 0.2	10.4 ± 0.5^	4914 ± 1655	∞

**Controls**						
DMH	4	100	26.1 ± 0.3	29.6 ± 1.4	186 ± 33	2.5 ± 0.5
Arc/ME/PT	4	100	23.7 ± 0.4	33.5 ± 1.2	253 ± 57	5.4 ± 1.1
SCN	4	100	24.3 ± 0.1	10.8 ± 0.5^	2408 ± 593	∞

^ phase for SCN cultures is in Zeitgeber time, not time from cull, as SCN rhythms are not reset by cull/culture procedure.

## Discussion

This study defines multiple circadian oscillators with different properties, in brain regions crucial for internal homeostatic regulation including the Arc, ME/PT and ependymal cell layer of the 3^rd ^ventricle and reveals, for the first time, endogenous rhythms in the DMH. Notably, we discriminated rhythms in single cells in the Arc and DMH, which were maintained following impairment of synaptic transmission, suggesting that cells in these structures have intrinsic pacemaking properties. Damping of whole tissue rhythms was due to both the desynchronization of these cellular oscillators, which drift out of phase over time reducing the coherent output of the tissue, and to the gradual damping in amplitude of individual oscillators. This is the first demonstration of single cell rhythms in PER2::LUC in any tissue outside the SCN, and we provide the first investigation of neurophysiological rhythms in the Arc and DMH. Both electrical and PER2 rhythms may gate the responses of cells in these regions rendering them more responsive to inputs at one time of day versus another. Importantly, we establish that overnight food deprivation alters the phase of PER2::LUC rhythms exclusively in the ME/PT, indicating that acute changes in metabolic status can differentially affect molecular clocks throughout the hypothalamus.

Diurnal rhythms in molecular clockworks have been previously noted in the ME/PT, Arc (reviewed in [16]) and ependymal cell layer of the 3^rd ^ventricle [48] using *in situ *hybridization and immunohistochemistry. These experimental procedures, however, reflect rhythmicity *in vivo *and can not directly address the capacity of a tissue to generate autonomous rhythms. Abe and colleagues demonstrated intrinsic rhythmicity of *per1 *mRNA expression in cultures of ME and Arc prepared from *per1::luc *rats, though such PMT recordings lack the capacity for precise anatomical or cellular localization of the oscillations [15]. Here, we localize endogenous PER2 expression to the ME/PT and ependymal cell layer and, importantly, discriminate single cells in the ArcD, ArcL and DMHc.

The ME/PT and ependymal cell layer are critical sites within the neuroendocrine system especially with regard to melatonin actions and photoperiodic timekeeping; indeed, there is good evidence that the PT is the site of a circannual pacemaker [30,49-54]. The ependymal cell layer contains a number of different cell types important in the regulation of metabolism, the movement of cerebrospinal fluid and control of hormonal exchange with the cerebrospinal fluid [55-57]. The PER2 rhythms observed here may drive expression of downstream genes and proteins and regulate the daily availability of hormones [58]. Likewise, the demonstrated circadian oscillators in the ME/PT may underlie some of the infradian timekeeping properties of these regions.

Importantly, we demonstrate that molecular rhythms in the ArcD are accompanied by circadian rhythms in spontaneous neuronal firing *in vitro*, in individual cells, in the absence of SCN input. These rhythms are of similar amplitude to those recorded in the SCN using the same 'open culture' experimental setup [40], but differ from the SCN in their longevity, where multiunit rhythms, although damping, can be monitored for up to 96 hours *in vitro*. Since most neural and metabolic inputs to the Arc are encoded and ultimately integrated and transmitted via changes in neuronal excitability, the circadian rhythm we observe in neuronal excitability in the Arc presumably provides a mechanism for communication of circadian information to downstream targets and indicates a significant functional role of circadian rhythmicity in this tissue.

Our data indicate, for the first time, that the DMH is intrinsically rhythmic. Previous investigations of *per2*/PER2 expression in the DMH *in vivo *under *ad libitum *feeding conditions revealed either a lack of expression [42], constitutively low expression [59-61] or very low amplitude rhythms [36]. Higher amplitude rhythms were observed *in vivo *under restricted feeding paradigms, but it was unclear if these resulted from inputs to the DMH from external oscillators or whether the tissue was capable of generating endogenous circadian rhythms [59]. This study reveals the ability of DMH neurons to generate endogenous rhythms. A number of possibilities may explain the discrepancies surrounding clock gene rhythmicity in the DMH *in vivo *and *in vitro*. Firstly, intrinsic rhythmicity in the DMH *in vivo *may be suppressed by the SCN, and it is only upon removal from this inhibition, via physical isolation from the SCN *in vitro*, that the endogenous pacemaker capability of the DMH is revealed. Secondly, oscillations in the DMH *in vivo *may be so low in amplitude as to make detection with discontinuous sampling techniques, such as immunohistochemistry or *in situ *hybridization, difficult. Thirdly, circadian rhythms in DMH neurons may only be inducible under metabolic duress, a state potentially reproduced by our culture environment.

The factors that underlie improved rhythmicity in the ArcD versus the ArcL and DMHc are unknown. A concurrent decrease in the synchronization and amplitude of individual cellular rhythms occurs over time, similar to the decreased amplitude observed in desynchronized SCN neurons in mice lacking the VPAC_2 _receptor [8,62]. These reports, along with our current data, indicate a correlation between synchronization of cells within a tissue and the amplitude of individual cellular rhythms, and highlight a key difference between SCN and MBH oscillators; the inability of MBH clock cells to maintain long term synchrony.

The SCN and MBH differ further in their phase-resetting properties after cull. The phase of peak PER2 expression in the SCN is correlated to ZT and not the time of cull, whereas in the Arc and DMH it is reset by the cull/culture procedure and consistently correlated with cull time (see additional file 1: Fig. S3). However, while the phase of *per1::luc *expression in the SCN does not correlate with time of cull, it is affected by it [63], although there is an epoch, during mid-late day at which the SCN is impervious to resetting by cull. The gating of responses to external stimuli is vital to the synchronization of oscillators with external zeitgebers. MBH oscillators presumably differ to the SCN in their gating/resetting properties rendering them more sensitive to resetting.

Studies in rodents and flies indicate an interdependence of molecular and cellular oscillators: damping of electrical firing or silencing of clock neurons attenuates clock gene rhythms [9,64]. The inhibition of sodium channel dependent cell-cell communication with TTX affects both the synchrony and amplitude of molecular oscillations of individual SCN neurons [9]. Here, however, we find no such dependence on electrical output of the amplitude of MBH molecular oscillations (see Fig. 6), and as MBH oscillators rapidly desynchronize even in the absence of TTX, the effects of this treatment on synchrony in the MBH are difficult to fully define. Nevertheless, our data also indicate a positive correlation between the strength of the molecular and cellular oscillators; the SCN shows the strongest degree of circadian rhythmicity in both measures, followed by the Arc, DMH then VMH. The extent of interdependence between molecular and cellular oscillators in the MBH is still to be fully resolved and the mechanisms linking them, both in these nuclei and in neural oscillators in general, are as yet unknown.

Fundamental differences between oscillators within the MBH are highlighted by the differential responses to metabolic challenge; only the phase of rhythms is altered in response to food deprivation, and only in the ME/PT. Our observation that overnight food deprivation does not alter DMH PER2 expression *in vitro *is in agreement with results obtained *in vivo *[36]. There are a wide variety of mechanisms through which altered metabolic states can be signaled to the circadian clockwork in a given tissue. For example, nuclear receptors and their co-activators, which respond to food intake and changing metabolic environments, are known to act directly on the circadian clockwork [2,24,65,66]. As in the forebrain or SCN, where NPAS2 can act as a functional analogue for CLOCK [67,68], it is feasible that the clockwork in the ME/PT may differ from that in other MBH regions in factors such as its core components and signaling mechanisms, in a way which renders it responsive to stimuli arising from food deprivation. This may selectively strengthen oscillations in the ME/PT, allowing this structure to maintain its *in vivo *phase while oscillations in the Arc and DMH are still reset by cull, as in control animals.

We observed no significant change in PER2::LUC expression in the MBH of HFF mice. This observation is in agreement with a previous investigation which found no significant change in the expression of *per2 *mRNA in the MBH in HFF mice [21]. HFF mice display alterations in the daily variations in various feeding peptides in the MBH, a damping of body temperature and locomotor activity rhythms in LD cycles, and a lengthening of the free-running period [21,47]. Despite this, we observed no feeding induced change in the expression of PER2 in the SCN. Taken together, these studies strongly suggest that neither SCN nor MBH expression of *per2 *mRNA or PER2 protein are involved in the circadian dysfunction associated with high dietary fat content. In contrast, HFF disrupts both the rhythmic expression of metabolic markers in the serum and circadian clock gene expression in fat and liver [21]. Thus it appears that peripheral clocks are more acutely sensitive to metabolic perturbations than central clocks. Further, the obesity-induced changes which occur in the daily expression of feeding peptides, such as increased leptin levels [47], are more likely to be associated with alterations in peripheral rather than either SCN or MBH oscillators.

## Conclusion

In conclusion, there are four main interrelated differences between MBH oscillators and the SCN. The MBH lacks the capability of the SCN to maintain cellular synchrony, it is reset by cull, it displays much lower amplitude molecular rhythms, and the robustness of cellular rhythms is decreased. Furthermore, MBH oscillators likely have different tissue specific properties and functions, indicated by their differential resetting following food deprivation. The intracellular and/or network properties enabling progressively tighter coordination of individual clock cells from ArcL through the DMHc and ArcD to the SCN remain to be determined, but conceivably include alterations in neurochemical signaling, cytoarchitecture, gap junctions or physical density of clock cells [69-71]. Future experiments on MBH oscillators will help to unravel the varying importance and interrelation of these circadian components on rhythm generation, and elucidate the significance of local timing in CNS tissues on the regulation of both tissue function and global circadian homeostasis.

## Abbreviations

Arc: arcuate nucleus of the hypothalamus; ArcD: dorsal arc; ArcL: lateral arc; DMH: dorsomedial nucleus of the hypothalamus; DMHc: pars compacta region of the DMH; FD: food deprived; HFF: high-fat fed; LD: light/dark; LUC: luciferase; MBH: mediobasal hypothalamus; ME: median eminence; PMT: photomultiplier tube; PT: pars tuberalis; SCN: suprachiasmatic nucleus; TTX: tetrodotoxin; VMH: ventromedial nucleus of the hypothalamus; ZT: Zeitgeber time.

## Competing interests

The authors declare that they have no competing interests.

## Authors' contributions

All authors conceived of and designed the study. Luminometry and photovideomicroscopy experiments to characterize MBH oscillators were performed and analyzed by CG and ATLH. Food deprivation experiments were performed and analyzed by CG and ATLH. High fat feeding experiments were performed and analyzed by CG and SN. TMB performed and analyzed electrophysiology experiments and designed and wrote software for data analysis. All authors contributed to writing and editing the manuscript and have read and approved the final manuscript.

## Supplementary Material

Additional file 1 **Figure S1. MBH rhythmicity is independent of serum in culture medium**. PMT recording of PER2::LUC expression in an MBH culture prepared in serum free, B27 containing medium. Addition of forskolin (10 μM) potently restarts rhythms in damped tissue, whereas medium change to control serum containing medium does not revive damped rhythms.
**Figure S2. Photograph of electrode positioning on the DMHc and ArcD for electrophysiological recordings**. Broken lines outline the glass suction electrodes, while component areas of the MBH are delineated in the lower panel as in Figure 1.
**Figure S3**. **The phase of peak PER2::LUC expression in Arc and DMH is reset by cull**. Rayleigh vector plots showing the phase of peak PER2::LUC expression *in vitro*, calculated as the time of first peak in bioluminescence after cull of animal, in the whole ArcD, ArcL and DMH regions for 12 cultures prepared at different times throughout the day/night. In all areas, the phase was significantly correlated with time of cull. Filled circles indicate the phase of peak bioluminescence for whole delineated ArcD, ArcL and DMH. The direction of the arrow indicates the mean phase vector and its length shows the significance of phase clustering, with the surrounding box indicating the variance of phase. The inner broken line indicates the significance threshold at p = 0.05.
**Figure S4. Raster plots of circadian PER2::LUC bioluminescence expression in individual cells in the ArcD (A), ArcL (B) and DMHc (C) from a single MBH slice**. Cells are stacked vertically, one cell per line; red indicates peak PER2::LUC emission, and green indicates minimal emission. Initially neuronal rhythms in PER2::LUC are synchronized then gradually drift out of phase and damp (shown by decreasing brightness on plot). 10 μM forskolin, added at 186 h, re-synchronizes circadian rhythms and increases the amplitude of oscillations of individual cells.
**Figure S5. Frequency histograms showing the periods of PER2::LUC expression of individual cells resolved in the SCN, ArcD, ArcL and DMHc**. SCN cells expressed the tightest range of periods, with most cells expressing a ~24 h period. Though cells of the ArcD, ArcL and DMHc expressed similar wide ranges of periods, the frequency distribution of cells in the ArcD peaked sharply at ~24 h whereas cells of the ArcL and DMHc expressed a more even distribution of periods across the range.
**Figure S6. Extracellularly detected electrical signals in MBH nuclei are dependent on sodium channel activity**. Multiunit (MUA; upper traces) and single unit activities (SUA; lower traces) detected in extracellular recordings from ArcD (**A**), DMHc (**B**) and VMH (**C**) are reversibly blocked by brief (5 min) applications of the Na^+ ^channel blocker tetrodotoxin (TTX; 0.5 μM).
**Figure S7: PMT recording of PER2::LUC expression in an MBH slice in the presence of 0.5 μM TTX**. Despite the presence of TTX, forskolin revived damped PER2::LUC rhythms.
**Figure S8: KCl stimulation restarts damped rhythms circadian rhythms**. PMT recordings of PER2::LUC expression in representative Arc/ME/PT (A) and MBH (B) slice cultures from food deprived animals. Addition of 10 μM KCl to the culture medium consistently restarted damped rhythms in all previously rhythmic MBH regions. Click here for file

Additional file 2 **Movie 1**. EM-CCD recording over 326 hours showing bioluminescence emission from an MBH slice culture prepared from a PER2::LUC mouse; one frame every 30 min. 10 μM forskolin was added to the slice at 186 h. Note the damping of cellular and tissue rhythms over the first 186 hours, which are revived by forskolin stimulation. Click here for file

Additional file 3 **Movie 2**. EM-CCD recording over 214 hours showing bioluminescence emission from an MBH slice culture prepared from a PER2::LUC mouse; one frame every hour. 10 μM forskolin was added to the slice at 164 h. Note the revival of individual cellular rhythms particularly in the DMHc. Click here for file

## References

[B1] Foster RG, Wulff K (2005). The rhythm of rest and excess. Nat Rev Neurosci.

[B2] Green CB, Takahashi JS, Bass J (2008). The meter of metabolism. Cell.

[B3] Laposky AD, Bass J, Kohsaka A, Turek FW (2008). Sleep and circadian rhythms: Key components in the regulation of energy metabolism. FEBS Lett.

[B4] Swerdlow A (2003). Shift work and breast cancer: a critical review of the epidemiological evidence. The Institute of Cancer Research, for the Health and Safety Executive.

[B5] Antle MC, Silver R (2005). Orchestrating time: arrangements of the brain circadian clock. Trends Neurosci.

[B6] Hastings MH, Herzog ED (2004). Clock genes, oscillators, and cellular networks in the suprachiasmatic nuclei. J Biol Rhythms.

[B7] Reppert SM, Weaver DR (2002). Coordination of circadian timing in mammals. Nature.

[B8] Maywood ES, Reddy AB, Wong GK, O'Neill JS, O'Brien JA, McMahon DG, Harmar AJ, Okamura H, Hastings MH (2006). Synchronization and maintenance of timekeeping in suprachiasmatic circadian clock cells by neuropeptidergic signaling. Curr Biol.

[B9] Yamaguchi S, Isejima H, Matsuo T, Okura R, Yagita K, Kobayashi M, Okamura H (2003). Synchronization of cellular clocks in the suprachiasmatic nucleus. Science.

[B10] Welsh DK, Logothetis DE, Meister M, Reppert SM (1995). Individual neurons dissociated from rat suprachiasmatic nucleus express independently phased circadian firing rhythms. Neuron.

[B11] Yamazaki S, Takahashi JS (2005). Real-time luminescence reporting of circadian gene expression in mammals. Methods Enzymol.

[B12] Schibler U, Ripperger J, Brown SA (2003). Peripheral circadian oscillators in mammals: time and food. J Biol Rhythms.

[B13] Brandstaetter R (2004). Circadian lessons from peripheral clocks: is the time of the mammalian pacemaker up?. Proc Natl Acad Sci USA.

[B14] Okamura H (2004). Clock genes in cell clocks: roles, actions, and mysteries. J Biol Rhythms.

[B15] Abe M, Herzog ED, Yamazaki S, Straume M, Tei H, Sakaki Y, Menaker M, Block GD (2002). Circadian rhythms in isolated brain regions. J Neurosci.

[B16] Guilding C, Piggins HD (2007). Challenging the omnipotence of the suprachiasmatic timekeeper: are circadian oscillators present throughout the mammalian brain?. Eur J Neurosci.

[B17] Hiler DJ, Bhattacherjee A, Yamazaki S, Tei H, Geusz ME (2008). Circadian mPer1 gene expression in mesencephalic trigeminal nucleus cultures. Brain Res.

[B18] Hastings MH, Maywood ES, Reddy AB (2008). Two decades of circadian time. J Neuroendocrinol.

[B19] Granados-Fuentes D, Saxena MT, Prolo LM, Aton SJ, Herzog ED (2004). Olfactory bulb neurons express functional, entrainable circadian rhythms. European Journal of Neuroscience.

[B20] Kalsbeek A, Kreier F, Fliers E, Sauerwein HP, Romijn JA, Buijs RM (2007). Minireview: Circadian control of metabolism by the suprachiasmatic nuclei. Endocrinology.

[B21] Kohsaka A, Laposky AD, Ramsey KM, Estrada C, Joshu C, Kobayashi Y, Turek FW, Bass J (2007). High-fat diet disrupts behavioral and molecular circadian rhythms in mice. Cell Metab.

[B22] Turek FW, Joshu C, Kohsaka A, Lin E, Ivanova G, McDearmon E, Laposky A, Losee-Olson S, Easton A, Jensen DR, Eckel RH, Takahashi JS, Bass J (2005). Obesity and metabolic syndrome in circadian Clock mutant mice. Science.

[B23] Rudic RD, McNamara P, Curtis AM, Boston RC, Panda S, Hogenesch JB, Fitzgerald GA (2004). BMAL1 and CLOCK, two essential components of the circadian clock, are involved in glucose homeostasis. PLoS Biol.

[B24] Liu C, Li S, Liu T, Borjigin J, Lin JD (2007). Transcriptional coactivator PGC-1alpha integrates the mammalian clock and energy metabolism. Nature.

[B25] Karlsson B, Knutsson A, Lindahl B (2001). Is there an association between shift work and having a metabolic syndrome? Results from a population based study of 27,485 people. Occup Environ Med.

[B26] Buijs RM, Kreier F (2006). The metabolic syndrome: a brain disease?. J Neuroendocrinol.

[B27] Bernardis LL, Bellinger LL (1998). The dorsomedial hypothalamic nucleus revisited: 1998 update. Proc Soc Exp Biol Med.

[B28] Sahu A (2004). Minireview: A hypothalamic role in energy balance with special emphasis on leptin. Endocrinology.

[B29] Choi S, Wong LS, Yamat C, Dallman MF (1998). Hypothalamic ventromedial nuclei amplify circadian rhythms: do they contain a food-entrained endogenous oscillator?. J Neurosci.

[B30] Duncan MJ (2007). Circannual prolactin rhythms: calendar-like timer revealed in the pituitary gland. Trends Endocrinol Metab.

[B31] Kalsbeek A, Palm IF, La Fleur SE, Scheer FA, Perreau-Lenz S, Ruiter M, Kreier F, Cailotto C, Buijs RM (2006). SCN outputs and the hypothalamic balance of life. J Biol Rhythms.

[B32] Sellix MT, Egli M, Poletini MO, McKee DT, Bosworth MD, Fitch CA, Freeman ME (2006). Anatomical and functional characterization of clock gene expression in neuroendocrine dopaminergic neurons. Am J Physiol Regul Integr Comp Physiol.

[B33] Gooley JJ, Schomer A, Saper CB (2006). The dorsomedial hypothalamic nucleus is critical for the expression of food-entrainable circadian rhythms. Nat Neurosci.

[B34] Landry GJ, Simon MM, Webb IC, Mistlberger RE (2006). Persistence of a behavioral food-anticipatory circadian rhythm following dorsomedial hypothalamic ablation in rats. Am J Physiol Regul Integr Comp Physiol.

[B35] Landry GJ, Yamakawa GR, Webb IC, Mear RJ, Mistlberger RE (2007). The dorsomedial hypothalamic nucleus is not necessary for the expression of circadian food-anticipatory activity in rats. J Biol Rhythms.

[B36] Moriya T (2009). The dorsomedial hypothalamic nucleus is not necessary for food-anticipatory circadian rhythms of behavior, temperature or clock gene expression in mice. European Journal of Neuroscience.

[B37] Yoo SH, Yamazaki S, Lowrey PL, Shimomura K, Ko CH, Buhr ED, Siepka SM, Hong HK, Oh WJ, Yoo OJ, Menaker M, Takahashi JS (2004). PERIOD2::LUCIFERASE real-time reporting of circadian dynamics reveals persistent circadian oscillations in mouse peripheral tissues. Proc Natl Acad Sci USA.

[B38] Paxinos G, Franklin KB (2001). The Mouse Brain in Stereotaxic Coordinates.

[B39] Brown TM, Hughes AT, Piggins HD (2005). Gastrin-releasing peptide promotes suprachiasmatic nuclei cellular rhythmicity in the absence of vasoactive intestinal polypeptide-VPAC2 receptor signaling. J Neurosci.

[B40] Brown TM, Banks JR, Piggins HD (2006). A novel suction electrode recording technique for monitoring circadian rhythms in single and multiunit discharge from brain slices. J Neurosci Methods.

[B41] Bechtold DA, Brown TM, Luckman SM, Piggins HD (2008). Metabolic rhythm abnormalities in mice lacking VIP-VPAC2 signaling. Am J Physiol Regul Integr Comp Physiol.

[B42] Feillet CA, Mendoza J, Albrecht U, Pevet P, Challet E (2007). Forebrain oscillators ticking with different clock hands. Mol Cell Neurosci.

[B43] Kubota A, Inouye ST, Kawamura H (1981). Reversal of multiunit activity within and outside the suprachiasmatic nucleus in the rat. Neurosci Lett.

[B44] Inouye ST (1983). Does the ventromedial hypothalamic nucleus contain a self-sustained circadian oscillator associated with periodic feedings?. Brain Res.

[B45] Kurumiya S, Kawamura H (1991). Damped oscillation of the lateral hypothalamic multineuronal activity synchronized to daily feeding schedules in rats with suprachiasmatic nucleus lesions. J Biol Rhythms.

[B46] Prolo LM, Takahashi JS, Herzog ED (2005). Circadian rhythm generation and entrainment in astrocytes. J Neurosci.

[B47] Mendoza J, Pevet P, Challet E (2008). High-fat feeding alters the clock synchronization to light. J Physiol.

[B48] Yasuo SC, von Gall, Weaver DR, Korf HW (2008). Rhythmic expression of clock genes in the ependymal cell layer of the third ventricle of rodents is independent of melatonin signaling. Eur J Neurosci.

[B49] Lincoln GA, Clarke IJ, Hut RA, Hazlerigg DG (2006). Characterizing a mammalian circannual pacemaker. Science.

[B50] Goldman BD (2001). Mammalian photoperiodic system: formal properties and neuroendocrine mechanisms of photoperiodic time measurement. J Biol Rhythms.

[B51] Dupre SM, Burt DW, Talbot R, Downing A, Mouzaki D, Waddington D, Malpaux B, Davis JR, Lincoln GA, Loudon AS (2008). Identification of melatonin-regulated genes in the ovine pituitary pars tuberalis, a target site for seasonal hormone control. Endocrinology.

[B52] Nakao N, Ono H, Yamamura T, Anraku T, Takagi T, Higashi K, Yasuo S, Katou Y, Kageyama S, Uno Y, Kasukawa T, Iigo M, Sharp PJ, Iwasawa A, Suzuki Y, Sugano S, Niimi T, Mizutani M, Namikawa T, Ebihara S, Ueda HR, Yoshimura T (2008). Thyrotrophin in the pars tuberalis triggers photoperiodic response. Nature.

[B53] Barrett P (2006). Photoperiodic regulation of cellular retinoic acid-binding protein 1, GPR50 and nestin in tanycytes of the third ventricle ependymal layer of the Siberian hamster. J Endocrinol.

[B54] Hanon EA, Lincoln GA, Fustin JM, Dardente H, Masson-Pevet M, Morgan PJ, Hazlerigg DG (2008). Ancestral TSH mechanism signals summer in a photoperiodic mammal. Curr Biol.

[B55] Bruni JE, Del Bigio MR, Clattenburg RE (1985). Ependyma: normal and pathological. A review of the literature. Brain Res.

[B56] Rodriguez EM, Blazquez JL, Pastor FE, Pelaez B, Pena P, Peruzzo B, Amat P (2005). Hypothalamic tanycytes: a key component of brain-endocrine interaction. Int Rev Cytol.

[B57] Tu HM, Kim SW, Salvatore D, Bartha T, Legradi G, Larsen PR, Lechan RM (1997). Regional distribution of type 2 thyroxine deiodinase messenger ribonucleic acid in rat hypothalamus and pituitary and its regulation by thyroid hormone. Endocrinology.

[B58] Devarajan K, Marchant EG, Rusak B (2005). Circadian and light regulation of oxytocin and parvalbumin protein levels in the ciliated ependymal layer of the third ventricle in the C57 mouse. Neuroscience.

[B59] Mieda M, Williams SC, Richardson JA, Tanaka K, Yanagisawa M (2006). The dorsomedial hypothalamic nucleus as a putative food-entrainable circadian pacemaker. Proc Natl Acad Sci USA.

[B60] Verwey M, Khoja Z, Stewart J, Amir S (2007). Differential regulation of the expression of Period2 protein in the limbic forebrain and dorsomedial hypothalamus by daily limited access to highly palatable food in food-deprived and free-fed rats. Neuroscience.

[B61] Verwey M, Lam GY, Amir S (2009). Circadian rhythms of PERIOD1 expression in the dorsomedial hypothalamic nucleus in the absence of entrained food-anticipatory activity rhythms in rats. Eur J Neurosci.

[B62] Hughes AT, Guilding C, Lennox L, Samuels RE, McMahon DG, Piggins HD (2008). Live imaging of altered period1 expression in the suprachiasmatic nuclei of Vipr2-/- mice. J Neurochem.

[B63] Yoshikawa T, Yamazaki S, Menaker M (2005). Effects of preparation time on phase of cultured tissues reveal complexity of circadian organization. J Biol Rhythms.

[B64] Nitabach MN, Blau J, Holmes TC (2002). Electrical silencing of Drosophila pacemaker neurons stops the free-running circadian clock. Cell.

[B65] Duez H, Staels B (2008). The nuclear receptors Rev-erbs and RORs integrate circadian rhythms and metabolism. Diab Vasc Dis Res.

[B66] Yang X, Downes M, Yu RT, Bookout AL, He W, Straume M, Mangelsdorf DJ, Evans RM (2006). Nuclear receptor expression links the circadian clock to metabolism. Cell.

[B67] Reick M, Garcia JA, Dudley C, McKnight SL (2001). NPAS2: an analog of clock operative in the mammalian forebrain. Science.

[B68] DeBruyne JP, Weaver DR, Reppert SM (2007). CLOCK and NPAS2 have overlapping roles in the suprachiasmatic circadian clock. Nat Neurosci.

[B69] Shirakawa T, Honma S, Honma K (2001). Multiple oscillators in the suprachiasmatic nucleus. Chronobiol Int.

[B70] Michel S, Colwell CS (2001). Cellular communication and coupling within the suprachiasmatic nucleus. Chronobiol Int.

[B71] Aton SJ, Herzog ED (2005). Come together, right.now: synchronization of rhythms in a mammalian circadian clock. Neuron.

