# Supplementary material for: A riot of rhythms: neuronal and glial circadian oscillators in the mediobasal hypothalamus
Source: Mol Brain. 2009 Aug 27;2:28. doi: 10.1186/1756-6606-2-28 (PMC2745382; doi:10.1186/1756-6606-2-28)
Supplement: Additional file 1 — Figure S1. MBH rhythmicity is independent of serum in culture medium. PMT recording of PER2::LUC expression in an MBH culture prepared in serum free, B27 containing medium. Addition of forskolin (10 μM) potently restarts rhythms in damped tissue, whereas medium change to control serum containing medium does not revive damped rhythms. Figure S2. Photograph of electrode positioning on the DMHc and ArcD for electrophysiological recordings. Broken lines outline the glass suction electrodes, while component areas of the MBH are delineated in the lower panel as in Figure 1. Figure S3. The phase of peak PER2::LUC expression in Arc and DMH is reset by cull. Rayleigh vector plots showing the phase of peak PER2::LUC expression in vitro, calculated as the time of first peak in bioluminescence after cull of animal, in the whole ArcD, ArcL and DMH regions for 12 cultures prepared at different times throughout the day/night. In all areas, the phase was significantly correlated with time of cull. Filled circles indicate the phase of peak bioluminescence for whole delineated ArcD, ArcL and DMH. The direction of the arrow indicates the mean phase vector and its length shows the significance of phase clustering, with the surrounding box indicating the variance of phase. The inner broken line indicates the significance threshold at p = 0.05. Figure S4. Raster plots of circadian PER2::LUC bioluminescence expression in individual cells in the ArcD (A), ArcL (B) and DMHc (C) from a single MBH slice. Cells are stacked vertically, one cell per line; red indicates peak PER2::LUC emission, and green indicates minimal emission. Initially neuronal rhythms in PER2::LUC are synchronized then gradually drift out of phase and damp (shown by decreasing brightness on plot). 10 μM forskolin, added at 186 h, re-synchronizes circadian rhythms and increases the amplitude of oscillations of individual cells. Figure S5. Frequency histograms showing the periods of PER2::LUC expression of individual cells r [file 1756-6606-2-28-S1.pdf]

Supplementary Figures

S1

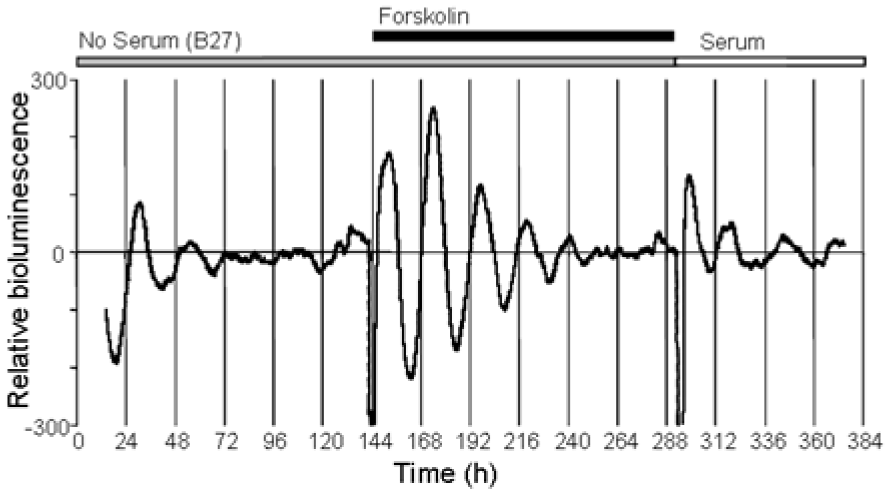

Supplementary Figures

S2

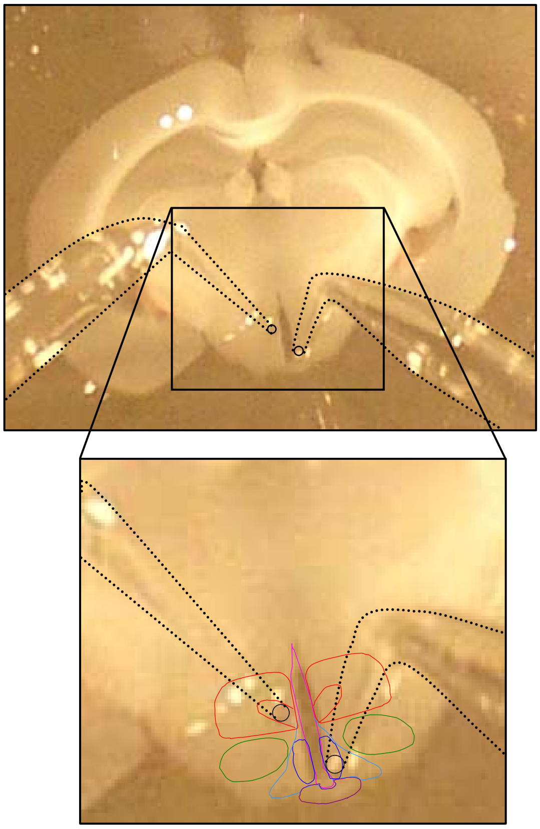

Supplementary Figures

S3

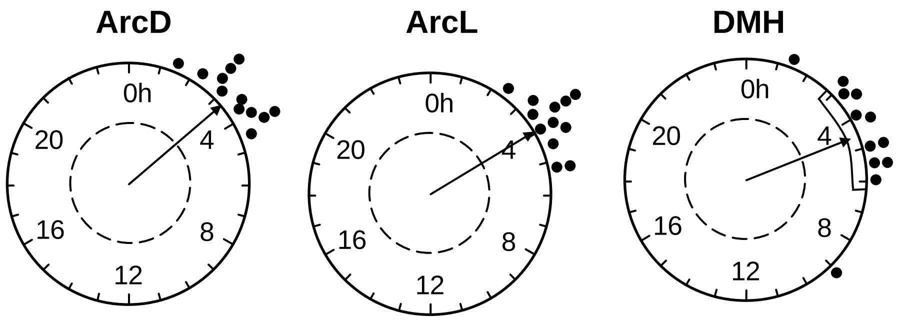

Supplementary Figures

S4

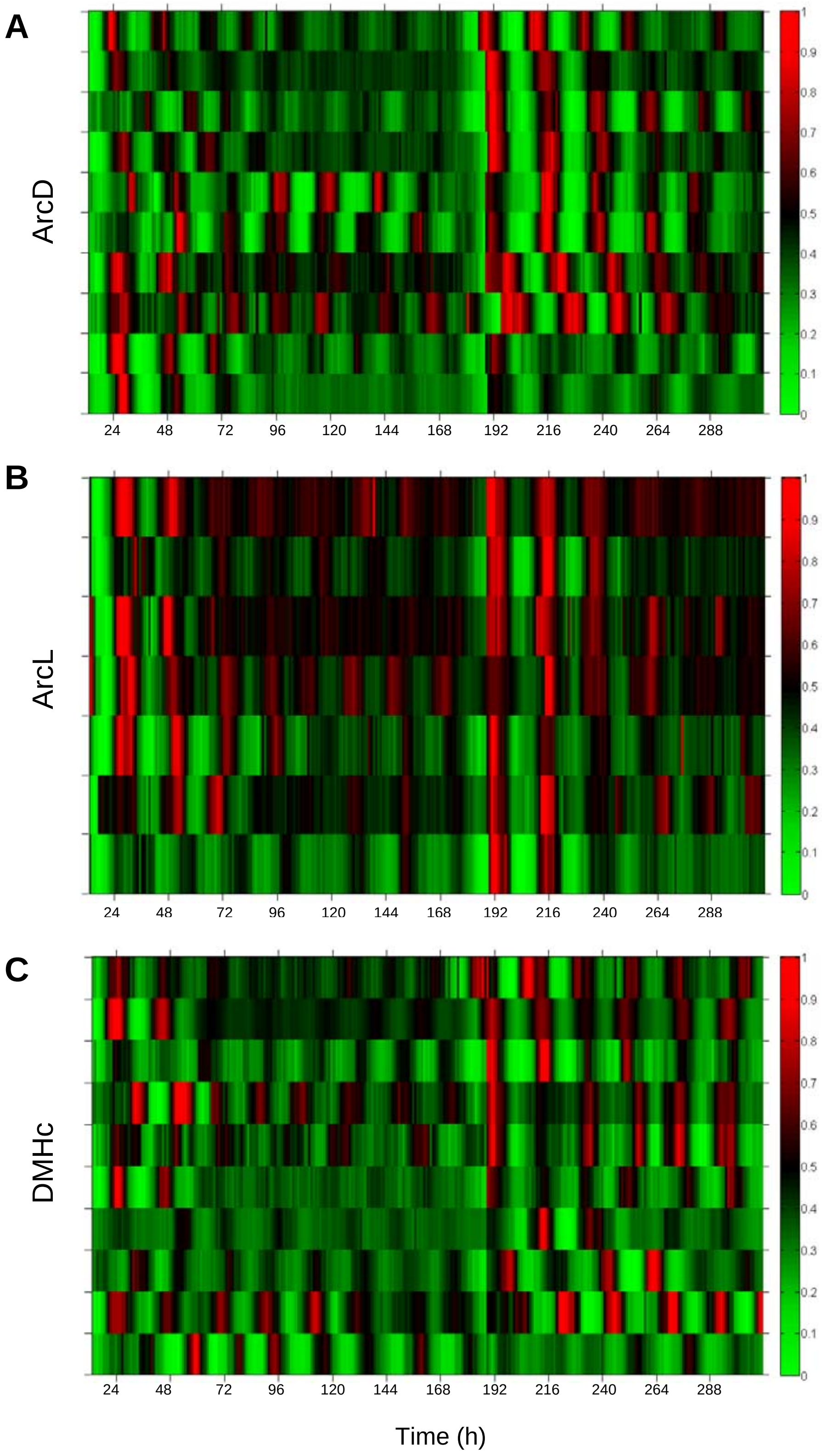

Supplementary Figures

S5

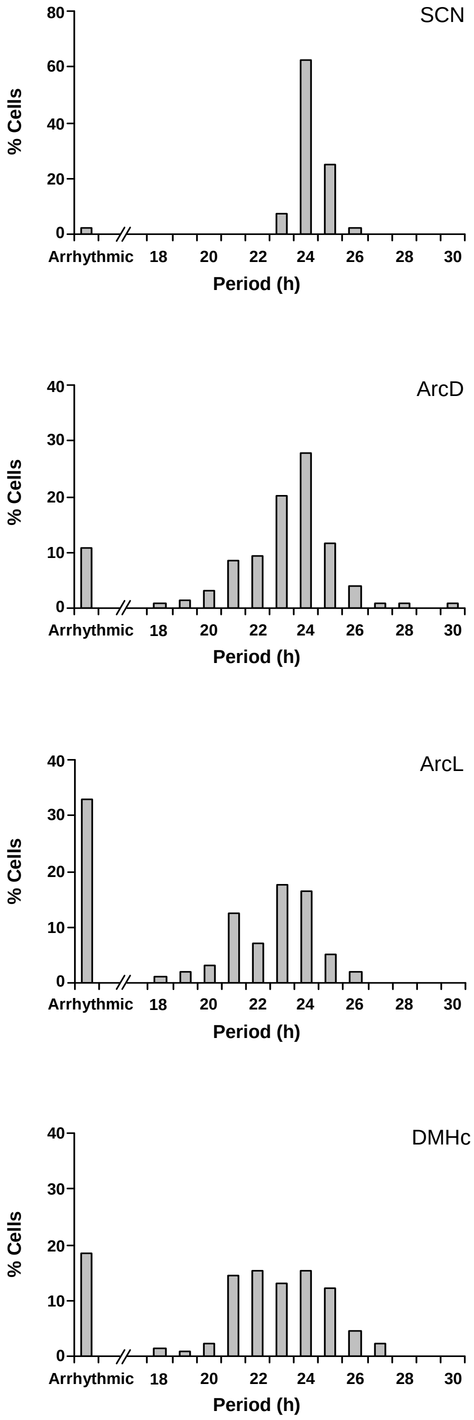

Supplementary Figures

S6

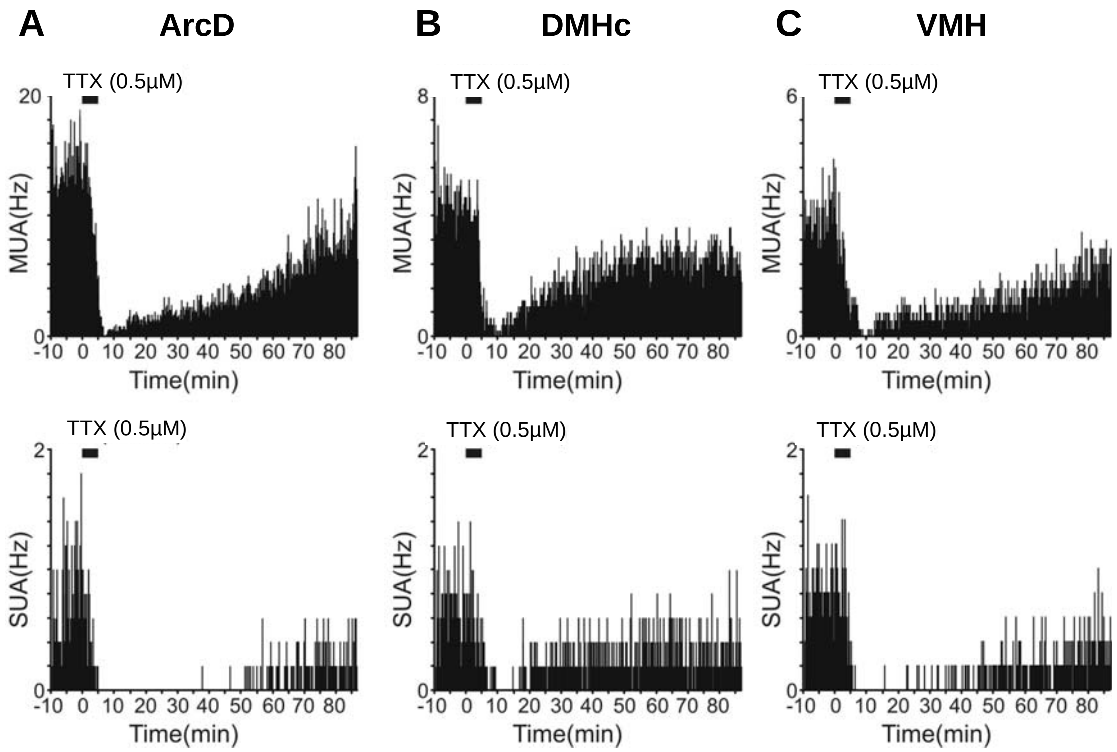

S7

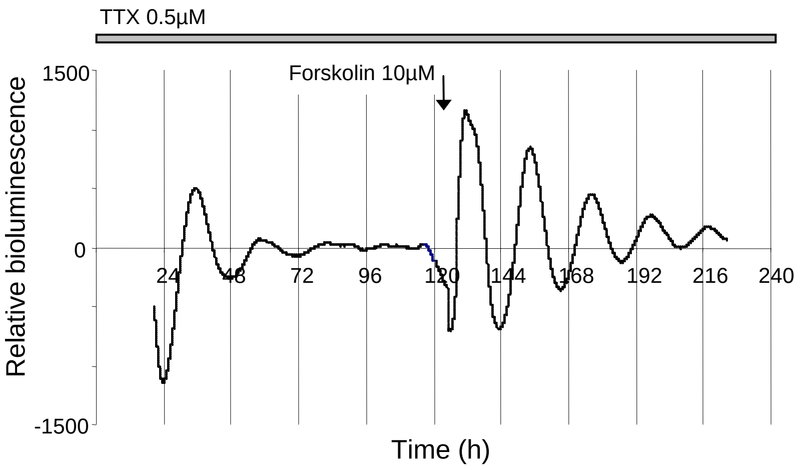

S8

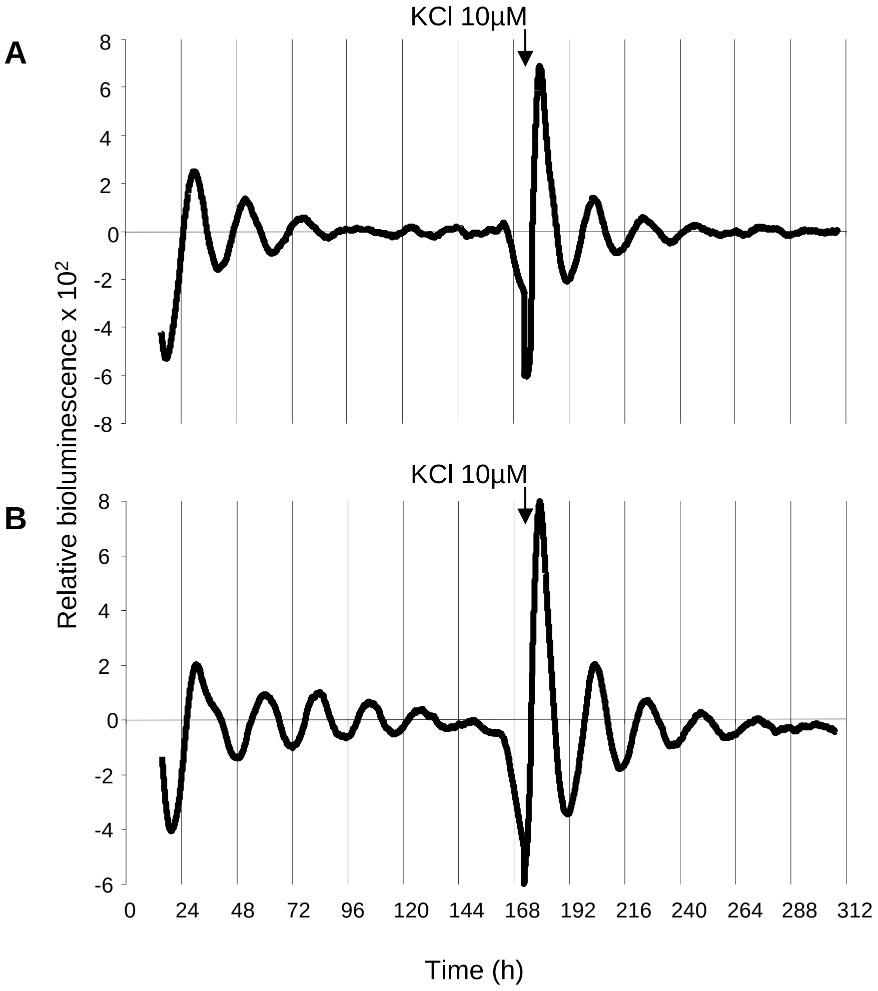
KCl 10µM
A
8
6
4
2
0
-2
-4
-6
-8
KCl 10µM
B
8
6
4
2
0
-2
-4
-6
Relative bioluminescence x 10^2
0
24
48
72
96
120
144
168
192
216
240
264
288
312
Time (h)
